# Creating a standardized, quantitative training protocol for upper limb bypass prostheses

**Published:** 2018-12-10

**Authors:** Conor Bloomer, Sophie Wang, Kimberly Kontson

**Affiliations:** 1 Center for Devices and Radiological Health, U.S. Food and Drug Administration, Silver Spring, MD, USA; 2 Department of Bioengineering, University of Maryland, College Park, USA

**Keywords:** activities of daily living, amputees, artificial limbs, learning, outcome assessment (health care), upper extremity

## Abstract

We aim to present a standard protocol for training able-bodied individuals to use a body-powered bypass prosthesis and assess training length and impact of prepositioning. The protocol design and subsequent analysis aims to facilitate controlled and efficient implementation of the able-bodied bypass user in the research setting. Six volunteers completed ten two-hour sessions with a body-powered bypass prosthesis. Each session included standardized training tasks: object manipulation, free training, and activities of daily living. Two outcome measures, a modified Southampton Hand Assessment Procedure and the Box and Blocks Test were used to score performance during each session. A standard learning curve was fitted to the scores to determine an optimal training length based on learning rate and learning plateau values; further tested through an effect size calculation. To assess prepositioning, scores were normalized and grouped by a measure of terminal device rotations. Scores then underwent a linear regression analysis. Optimal training lengths were found to be three and six sessions for modified Southampton Hand Assessment Procedure and Box and Blocks Test results respectively, with support from effect size calculations. Prepositioning and normalized score were weakly correlated, +0.38, and poorly fit, *R*^*2*^ = 0.016, contradictory to the expected strong correlation that would accompany the supposed performance benefits attributed to prepositioning. A lack of resources to guide the use of upper limb bypass prostheses is addressed with the presented standard, quantitatively assessed protocol. A framework for evaluating adequate training length and prepositioning is established and shared.

## Introduction

In upper limb amputee and prosthesis research, the bypass prosthesis has shown promise as a resource in study design [1–5]. A bypass prosthesis, or prosthetic simulator, is a device that allows a non-disabled user to activate a specific type of terminal device with similar controls that an amputee would use to operate a custom-made prosthesis. This allows upper limb prosthesis research to be conducted without the challenges of recruiting from a small population of upper limb amputees with varied prosthesis experience. Instead, by recruiting from the able-bodied population, larger samples can be more easily achieved and subject experience more readily controlled [5]. Additionally, previous evidence has shown similarities between bypass users and amputee users in kinematic profiles, visuomotor behaviours, and perceptual experiences, all lending credibility to the use of such devices as surrogates for amputee users [6–9]. However, to fully take advantage of a prosthesis’s capabilities and generate valid comparative results, able-bodied participants need adequate training. In contrast, to ensure feasibility of research with bypass users, training needs be both controlled and efficient. This need presents a unique challenge for researchers using these devices as they aim to control and justify distinct aspects of training within their own experimental designs and protocols.

This practical barrier to utilizing bypass prostheses has been addressed with some success in previous studies [1,3,10]. The selection of appropriate training content for example, largely focused on repetition of common tasks, is well established across studies and is supported by diverse work in the fields of motor learning and skill acquisition [11]. In bypass training specifically, these tasks include basic object manipulation, such as those demonstrated by Huinink (2016), and more complicated activities of daily living (ADLs), commonly drawn from the rehabilitation space [12–14]. The ordering of such tasks within a training regimen has additionally been investigated. Previous work has shown not only the ordering of task types, but the use of random vs blocked trials can be optimized to improve skill acquisition and inform training protocols [1,3].

However, training methodologies in the bypass literature continue to vary extensively in other regards, restricting the role of the bypass as a surrogate for amputee users to the specific context of the training used. The length of training, for example, may range from one minute to several weeks in existing bypass protocols [3, 5]. Not surprisingly, performance with a prosthesis has been shown to improve with training, therefore limiting variability in the amount of training may be a critical control [10]. Looking to the clinical space similar variability exists, with recommended ranges extending from five hours up to several months [12,15]. Agreement concerning training length in both spaces may be limited in part by the current reliance on subjective, qualitative measures. This trend can be readily seen in the Veterans Affairs and Department of Defense’s joint 2014 Management of Upper Extremity Amputation Rehabilitation guideline where only one of 27 concluding recommendations was empirically based; the remaining were supported by expert opinion [14]. To overcome this, a variety of objective outcome measures (i.e. Jebsen Taylor Hand Function Test, Southampton Hand Assessment Procedure, Box and Blocks Test, etc) have been used to assess ability across a rehabilitation regimen, with recent efforts specific to upper limb prosthesis use demonstrated in the literature [16–18]. However, the use of these measures to define a training endpoint and consequently assess training length has yet to be investigated in the specific context of bypass users.

In addition to training length, some other aspects remain variable across bypass studies. The role of coaching, for example, has been demonstrated to increase training gains compared to independent practice [10]. One frequently recommended coaching point is the need for prepositioning. In a common terminal device, such as the Hosmer hook used in this study, manual rotation is possible, restoring one degree of freedom lost with the wrist. Manual prepositioning, rotating the terminal device into an optimal orientation before attempting a task, is believed to greatly assist in successful action [10,12,15]. However, a controlled mean of introducing this concept in the bypass case, or quantitative support of its impact, hasn’t been presented previously.

Addressing the variability and limited resources in prosthesis training, this paper presents a standard training protocol for upper limb bypass prostheses. Aspects such as content and ordering are proactively controlled based on evidence in the existing literature and explicitly described to facilitate future use. Aspects with less agreement, namely training length and prepositioning, are assessed longitudinally through quantitative outcome measures to generate evidence to inform their future standardization in training protocols. This novel protocol may serve as a foundation to support and facilitate more widespread use of bypass prostheses and reduce the recruitment and methodological barriers that may limit a greater expanse of upper limb prosthesis research in general.

## Methods

### Participants

Six subjects (three female, three male; mean age 28.67 ± 3.27) with no upper limb disability or impairment were asked to complete ten two-hour training sessions, accumulating 20 hours of training each, or 60 total training sessions across the entire sample. This sample size was used due to the longitudinal commitment required as well as the large amount of data gathered from each subject in addressing our experimental aims. All subjects were right hand dominant per the Edinburgh handedness inventory [19]. The study was approved by the Institutional Review Board (Research Involving Human Subjects Committee) of the U.S. Food and Drug Administration (RIHSC #14–086R). All subjects provided written informed consent prior to participating in the study.

### Bypass prosthesis

**Figure 1.**
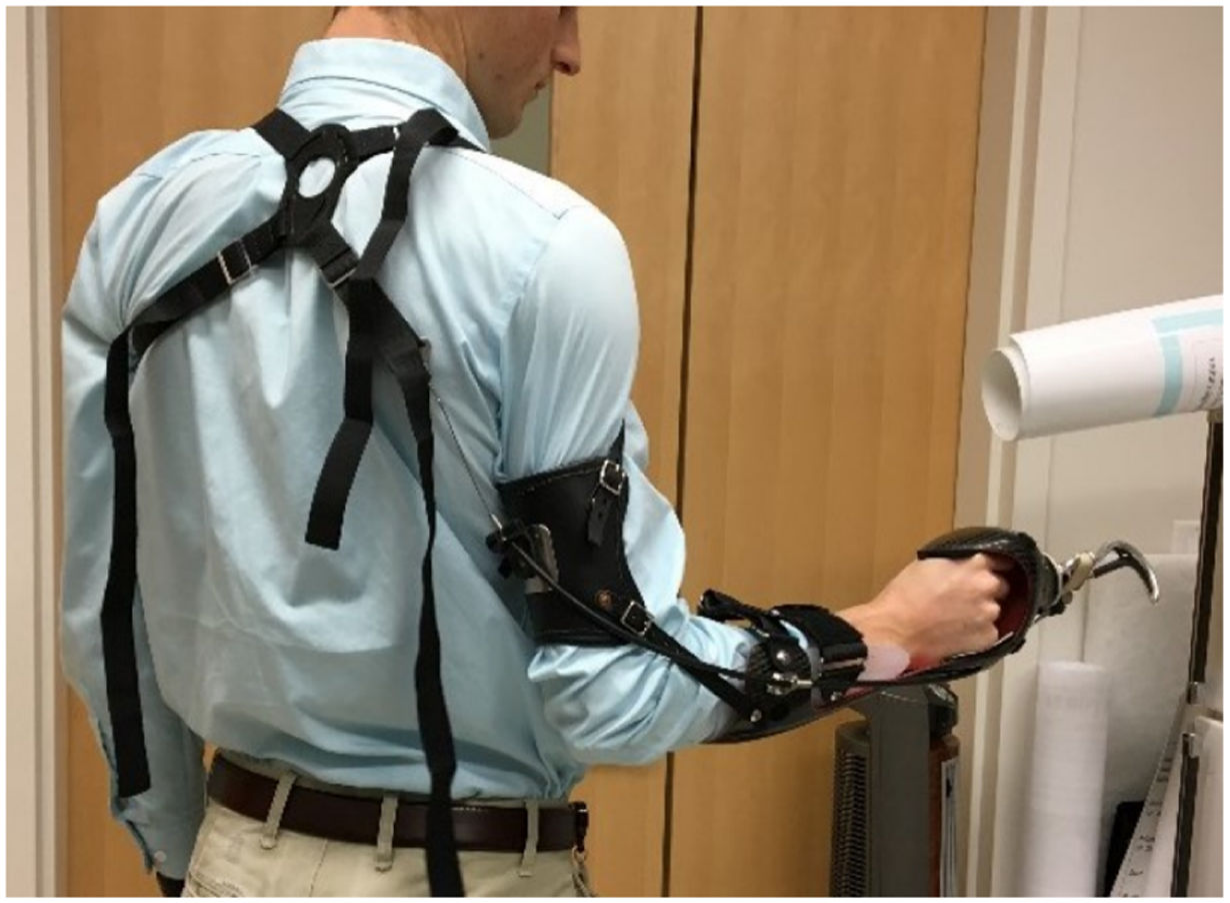
Donned body-powered bypass prosthesis

Arm Dynamics (Dallas, TX) provided the body-powered bypass prosthesis, featuring a voluntary opening Hosmer 5X hook terminal device, with manual wrist rotation possible, and a figure eight harness mechanism (Figure 1). The bypass prosthesis was designed with a distal offset of 12 cm.

### Training

**Figure 2.**
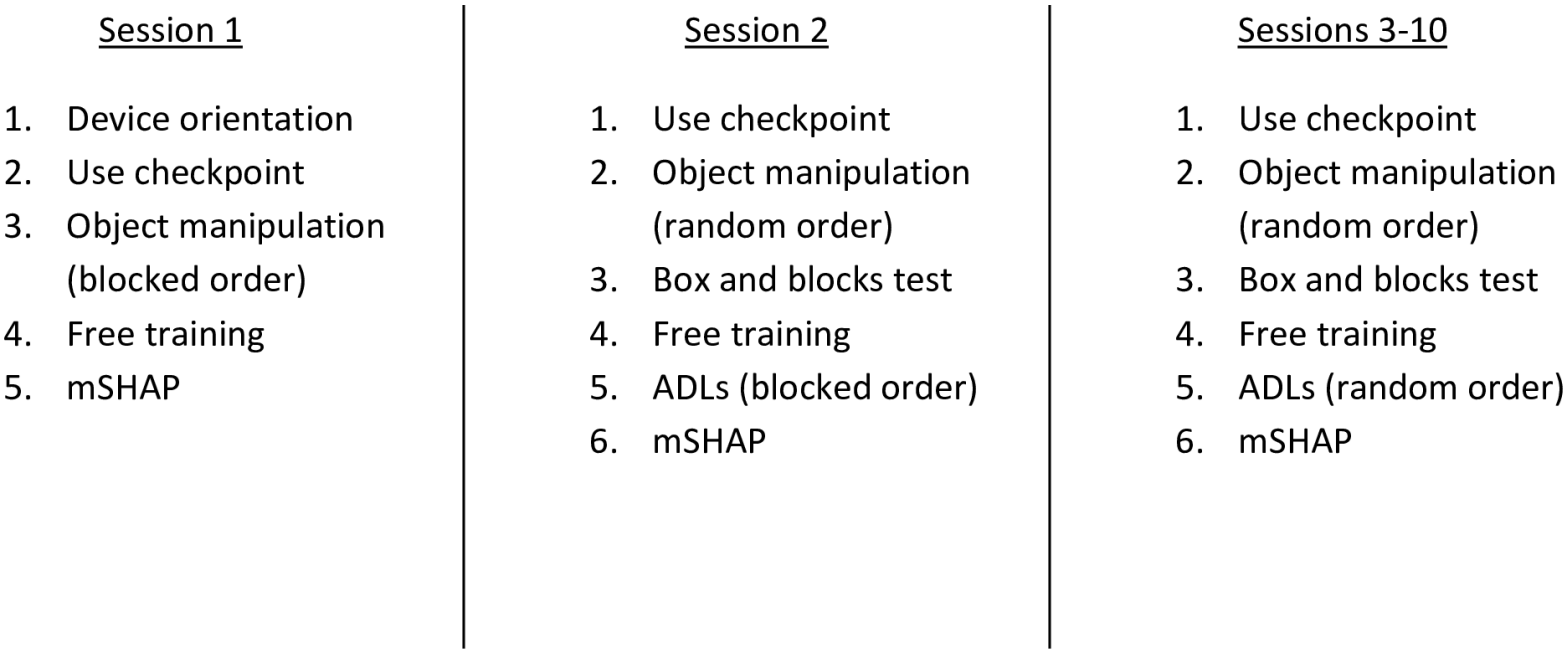
The order of each category of training task and outcome measure within the protocol

Training sessions consisted of a set of standardized tasks with variety only in their order and number of trials. The first session included a brief orientation covering device operation, followed by a verbal quiz on orientation content, and finally standard object manipulation tasks and free training. Subsequent sessions featured the same training activities with the addition of ADLs per the structure shown in Figure 2. The complete protocol with task descriptions, materials, and scripts can be found in the Protocol S1. Subjects were asked to participate in two to three sessions per week, with extended time allowed between sessions five and six to avoid fatigue. However, to ensure retainment, scheduling was not strictly controlled resulting in an average of 5.29 ± 5.91 days between sessions (1–5 and 6–10) and an average of 46.00 ± 17.96 days between sessions five and six. The variability in the timing of the training sessions was due to subject scheduling conflicts. However, we note that this type of recruitment and scheduling issue may be more representative of the barriers encountered when implementing a standardized training program for bypass prosthesis use in the lab setting.

Standardized training in this protocol began with object manipulation tasks, a category of simple grasping tasks that are widely used in rehabilitation practice [12,14,15]. Specifically, for prosthesis users, three types of object manipulations have been described, namely direct grasping (DG), indirect grasping (IG), and fixation (FIX). Respectively, these interactions describe unassisted unilateral grasp with the prosthesis, unilateral prosthesis grasp with assistance and transfer from the sound hand, and non-grasp unilateral fixation between the terminal device and the object in question [20]. Task descriptions and supporting evidence in the specific bypass prosthesis case have been demonstrated in previous work by Huinink (2016) informing the inclusion of such tasks in this protocol [3]. Subjects were asked to transport three distinct objects (wood cube, metal can, foam ball) using both direct grasping and indirect grasping and to complete three distinct tasks (opening/closing a zipper, using a ruler, undoing buttons) using fixation with the prosthesis.

Free training allowed subjects unstructured time to use the bypass prosthesis in both seated and standing positions for five minutes each. This portion of the training served several purposes. It encouraged subjects to experiment with motions and activities individually, facilitating greater personal confidence with the device and maintenance of some natural variability between users [21]. We also wanted to maintain subject engagement throughout the session by providing time with more freedom to pursue tasks of interest within the limitations of the controlled setting. The inclusion of both sitting and standing portions was meant to increase variety, mimicking more closely the experience of amputee users. Subjects were limited to the space around a table featuring several objects (e.g. pens, magazine, coins, etc.) and games (e.g. Perfection^®^, Rubik’s Cube^®^, Jenga^®^, etc.) and were instructed to make use of the bypass prosthesis during the 10-minute period.

Finally, ten ADL tasks were performed during a 20-minute period. ADL tasks have long been used in rehabilitation to train amputees and have been included in multiple training studies [2,10,21]. From multiple collections of listed ADLs, eight were selected specifically from the Activities Measure for Upper Limb Amputees, for their explicit instructions and reproducibility as well as their strength as part of a validated outcome measure [22]. An additional two tasks were developed by the research team based on existing lists of recommended ADLs [12–14,22]. The 20-minute period was used, as opposed to the trial completion format in the object manipulation tasks, to allow for greater flexibility in training on the more complicated ADL tasks while ensuring the overall timing of the session was maintained.

The ordering of tasks progressed from blocked order to random order formats within each category; object manipulation tasks and ADLs. This was done based on specific evidence of increased acquisition initially with consistent repetition and later with increased contextual interference [1]. In the object manipulation section, four trials for each task in the blocked order was reduced to two trials when randomized to accommodate the additional time taken when transitioning between tasks more frequently. In the ADL section a time limit was already imposed at 20 minutes therefore two trials were maintained for both blocked order and random order.

### Measurements

To assess the impact of training within our protocol two outcome measures, a modified Southampton Hand Assessment Procedure (mSHAP) and the Box and Blocks Test (BBT), were used. These outcome measures were administered during each training session for each subject, resulting in a total of 60 mSHAP scores and 50 averaged BBT scores, as a function of time and subject. All tasks times (mSHAP) and number of blocks transported (BBT) for each session and subject can be found in Tables S1-S7.

#### Modified Southampton Hand Assessment Procedure (mSHAP):

The mSHAP was administered once during each session. Participants were informed of the instructions and allowed one minute to practice prior to each task. Participants could adjust the orientation of the terminal device before each task. One attempt was allowed. A “Did Not Finish” result was recorded for unsuccessful attempts (e.g. an object was knocked off the table).

The mSHAP consists of 22 of the original 26 tasks outlined by the Southampton Hand Assessment Procedure. Four tasks (“Remove Jar Lid”, “Food Cutting”, “Pick Up Coins”, and “Lifting a Tray”) were not administered. The first two tasks were eliminated due to an inability to complete the tasks when using the bypass prosthesis during initial testing. The third and fourth tasks were eliminated to maintain the ecologically valid representation of prehensile patterns claimed by the Southampton Hand Assessment Procedure [23]. Using a normative data set, boundary conditions of the average normative time (normal function) and eight times the average normative time (minimal function) were applied to each task and times were scaled from zero to one hundred, where *T*_*s*_ is the scaled ‘task score’, *m* is the upper boundary, and *n* is the lower boundary. Task scores were calculated (*Ts*_*task name*_) were calculated for each task, shown in Table 1.

(1) $$T_{s}(t)=\frac{m-t}{m*n}*100$$

Unsuccessful attempts were automatically scaled to zero. The task scores were then grouped into linear indexes of functionality (*LIFs*) for each prehensile pattern: spherical, tripod, power, lateral, tip, and extension [23].

(2) $$LIF_{pp}=\frac{1}{k}\sum_{j=1}^{k}T_{sj}$$

In this equation, *k* is the sum number of tasks associated with a prehensile pattern, *pp*, and *j* represents one task within the grouping. Finally, a summary score weighting each *LIF*_*pp*_ by its number of associated tasks was calculated, generating a weighted linear index of functionality (*wLIF)* [24].

(3) $$wLIF\;=\;\frac{1}{25}\left(3LIF_{spherical}+3LIF_{tripod}+6LIF_{power}+5LIF_{lateral}+5LIF_{trip}+3LIF_{extension}\right)$$

#### Box and Blocks Test (BBT):

Three trials of the BBT were administered on sessions 2–10 (total of 27 trials per subject). The test was administered following performance of object manipulation tasks. Participants were informed of the instructions and allowed one 15-second practice period. Time was also allotted for participants to rest and to adjust the orientation of the terminal device before each trial.

The BBT consists of one hundred and fifty 2.5cm^3^ blocks distributed randomly on one side of a partition within a box. Participants were instructed to transport the blocks unilaterally with the bypass prosthesis as quickly as possible across the 15.2 cm tall partition, with the terminal device crossing the partition for each block transport. The participants score was equal to the number of blocks successfully transported in one minute [25].

### Analysis

#### Determining training length:

Scores from each outcome measure (individual *wLIF* and BBT; mean *wLIF* and BBT; and mean *T*_*s*_ and *LIF*_*pp*_) were fit to an inverse curve using nonlinear regression, defining a standard learning curve model [26]. From the fitted curve, two characteristic values were generated defining the asymptote, or learning plateau, (*a*) and the initial slope, or learning rate, (*b*). The following equation was used, Y = a − bX where *Y* is the outcome measure scores and *X* is the session number. Using *Y = 0.9a*, 90% of the subject’s plateau, as a training endpoint, an estimate of an adequate number of training sessions was calculated accordingly [26]. *R*^*2*^ values we’re used to evaluate the goodness of fit of the model to qualify results. While overall training length results come from *wLIF* and BBT scores, analysis of *T*_*s*_ and *LIF*_*pp*_ scores were also included to assess the individual components that contributed to these results. Specifically, *LIF*_*pp*_ data was included, despite the single grip of the Hosmer hook, to assess differences in performance on grip-oriented tasks that could be used to inform future training. *T*_*s*_ data was similarly included to assess individual task performance.

In a secondary analysis on average outcome measure scores, effect size was calculated to assess the impact of each session on measured performance. Effect size, as used here, compares the difference in scores between sessions normalized by the standard deviation between these differences per the following equation.

(4) $$ES=\frac{\;mean\;{\left(wLIF_{i}-wLIF_{i-1}\right)}_{total\text{ group}}}{\;std\;{\left(wLIF_{i}-wLIF_{i-1}\right)}_{total\text{ group}}}$$

In this equation, *i* represents the session number beginning with the second session [27]. A negative effect size represents a decrease in performance with the additional session, while positive results above 0.20, 0.50, 0.80 represent small, medium, and large effects, respectively [28].

#### Evaluating prepositioning:

**Figure 3.**
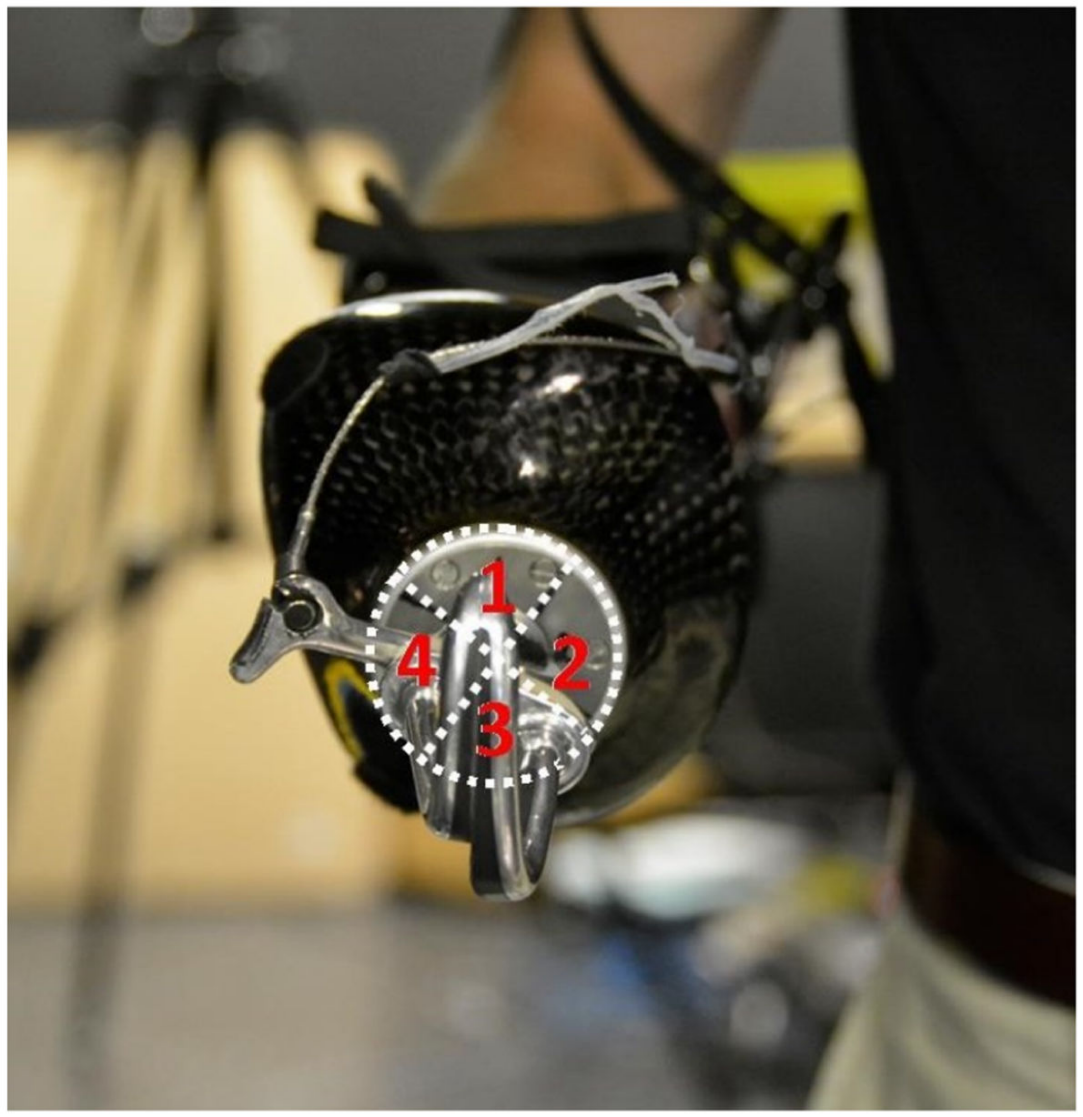
Boundaries and labelling of the quadrant system used to define terminal device orientation superimposed over the bypass terminal end

The orientation of the terminal device used for each mSHAP task was recorded per the system shown in Figure 3. The possible 360° of the terminal devices was partitioned into four equal quadrants. The orientation of the hook was defined by which quadrant the tips of the hook pointed to. Comparing the recorded quadrants for each task the number of orientation changes throughout the assessment could be gathered and used as an indirect measure of a participants’ utilization of the prepositioning strategy.

To assess whether additional quadrant changes (i.e. increased prepositioning) led to higher than average scores, the following analysis was done. The *wLIF* of each subject, *i*, for a given session, *j*, was normalized by subtracting the mean of that session, *wLIF*_*avg,j*_. *M* represents the total number of subjects.

(5) $$wLIF_{norm_{i,j}}=wLIF_{i,j}-wLIF_{\alpha vg,j}$$

(6) $$wLIF_{\;avg,j}=\frac{\overset{M}{\underset{i=1}{\sum }}wLIF_{i,j}}{M}$$

Next, a vector *Q*_*i,j*_ was calculated to represent the number of times the *i*^*th*^ subject changed the orientation of the hook to a different quadrant during the *j*^*th*^ session; each *Q*_*i,j*_ had a corresponding *wLIF*_*normi,j*_ variable. All *wLIF*_*normi,j*_ values with the same *Q*_*i,j*_ were grouped together, then averaged (Eq. 7). In the equation below *n* represents the number of wLIF_normi,j_ scores with a Q group.

(7) $$wLIF_{avg,Q}={\left[\frac{1}{n}\left(\sum_{i=1}^{n}wLIF_{norm}\right)\right]}_{Q}$$

A linear regression analysis was then performed on these values, plotted *Q*_*ij*_ vs *wLIF*_*avg,Q*_ to determine whether a positive correlation was present between prepositioning and measured performance. Individual quadrant change counts can be found in Supplementary Table 8.

## Results

### Determining training length

#### Modified Southampton Hand Assessment Procedure (mSHAP):

**Figure 4.**
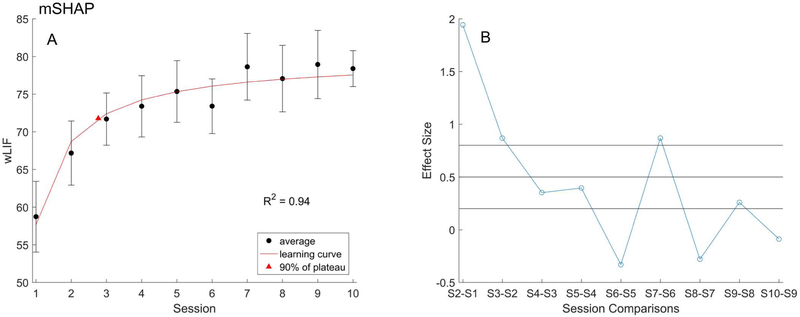
(A) The learning curve model (red) fit to the mean *wLIF* with standard error (black). Training endpoint per the model, at 90% of the plateau value, is marked (triangle). (B) Effect size (blue) for each successive session, thresholds (black) at 0.8, 0.5, and 0.2-mark minimum values for large, medium, and small effects respectively

A learning curve model was fitted to the *wLIF* data (Figure 4A) defined by characteristic values: learning plateau (*a*), learning rate (*b*), *R*^*2*^ (goodness of fit), and training endpoint (90% of plateau session). The learning rate was 22.09, which represents the slope of the initial gains in the curve, or an average increase of 22% of function during this phase. The learning plateau was 79.75, which represents the projected average ceiling of function with the prosthesis. The *R*^*2*^ value was 0.94, which supports our original learning curve assumption and our subsequent outputs. Finally, using 90% of the learning plateau as an efficient training endpoint the corresponding training length was found to be three sessions.

Effect sizes were generally in agreement, excluding the S7-S6 data point, and confirmed the greatest efficacy of training in the first three sessions (Figure 4B). Effects for sessions two and three we’re both large at 1.942 and 0.8679, respectively. Following the third session, effect sizes were small, below 0.5, or even negative. Individual *LIF*_*pp*_ and *T*_*s*_ values were similarly analysed to determine any differences in the learning of particular skills or tasks, with values shown in Table 1. Amongst prehensile patterns, the average *R*^*2*^ value was 0.75 ± 0.14. All six prehensile patterns R^2^ values exceeded 0.4, indicating moderate to strong correlations. Additionally, *LIFpp* demonstrated at most five sessions to reach the 0.9*a* endpoint; the lateral grip only requiring two sessions.

For *T*_*s*_ 12 of the 22 tasks had R^2^ values greater than 0.4. Of those 12 only one required more than five sessions to achieve the endpoint, with an average of 4.08 ± 1.24 sessions needed. For these skills and tasks, additional training across the whole ten session regimen appears to have little impact. Across all tasks, the average R^2^ value was 0.43 ± 0.33. In contrast to *LIF*_*pp*_ values the poorest *T*_*s*_ fit was as low as 0.005, discounting the characterization of this particular task (heavy spherical) by a learning curve model.

#### Box and Blocks Test (BBT):

**Figure 5.**
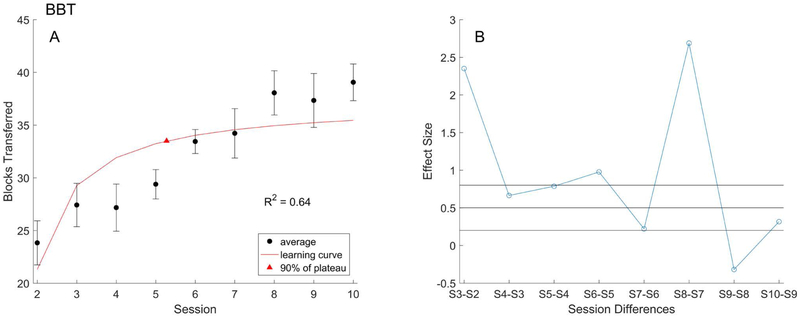
(A) The learning curve model (red) fit to the mean BBT scores with standard error (black). Training endpoint per the model, at 90% of the plateau value, is marked (triangle). (B) Effect size (blue) for each successive session, thresholds (black) at 0.8, 0.5, and 0.2-mark minimum values for large, medium, and small effects respectively

The learning curve model was fit in the same way to mean BBT scores, shown in Figure 5A. The learning rate was 15.91, representing an initial average improvement of 15.91 blocks during the rapid acquisition phase. The learning plateau was 37.21. The *R*^*2*^ value was 0.64, supporting the characterization of BBT progression as a learning curve, although not as strongly as the mSHAP. Ultimately, six sessions were needed to reach our training endpoint of 90% of learning plateau. This length contrasts that of the mSHAP data, but still falls well short of the experimental ten session regimen.

Effect sizes generally support the above conclusion, with large to medium effects seen through session six (Figure 5B). The following sessions, excluding S8-S7, had small or negative effects.

### Evaluating prepositioning

**Figure 6.**
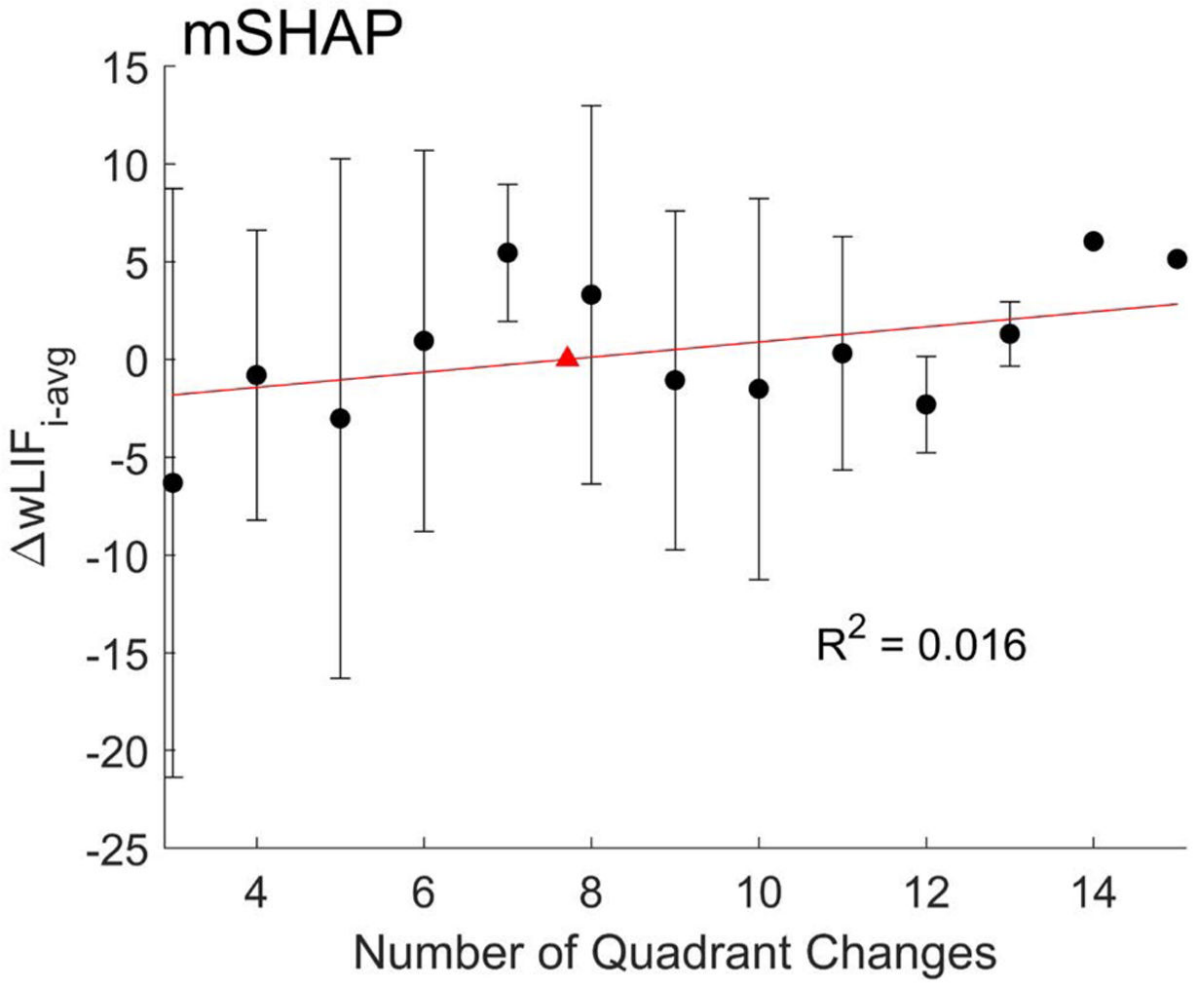
A linear model (red) fit to the mean Δ*wLIFi-avg* for each number of quadrant changes used per mSHAP completion with standard error (black)

A linear regression analysis was performed on calculations of *wLIF*_*avg,Q*_ vs *Q*_*i,j*_ to determine whether the expected positive correlation between prepositioning and performance existed, shown in figure 6. The linear fit showed a weak positive correlation, suggesting some minimal improvement with additional prepositioning, however with a poor *R*^*2*^ value of 0.016. This is contrary to the existing training recommendations that would expect clearer improvements with prepositioning. Observing the poor fit of the linear model, and the parabolic trend around the peak mean, an optimal amount of prepositioning may be more likely.

## Discussion

Not without limitations, our presented protocol successfully demonstrated and explicitly described a novel standardized training format for upper-limb prosthesis researchers employing a bypass device. Importantly, the inclusion of quantitative measures gives context to future training decisions and more clearly demonstrates strengths and weaknesses of both accepted methods and our own additions. As a framework, this protocol can facilitate the use of upper limb bypass prostheses in future studies, as well as stand as a foundation for further analysis of training decisions and formats.

With supporting results from both goodness of fit and effect size calculations we were able to demonstrate a uniquely efficient training endpoint through our learning curve analysis. However, results are tempered by unexplained outlying values at S7-S6 and S8-S7 exist for both mSHAP and BBT effect sizes respectively. The unexpected peak in the mSHAP analysis may be explained by the extended time off between sessions five and six; 46.00 ± 17.96 days versus 5.29 ± 5.91 days for all other sessions. This gap may have led to worse performance during session six inflating effect size at session seven as performance was recovered. However, this would not explain the same trend seen in the BBT data shifted one session further, requiring future work to explain. While we chose to maintain a flexible schedule to accommodate subjects, future work with stricter scheduling controls would be crucial in assessing spacing effects and further optimizing any protocol.

Not limited to effect size, we also demonstrated differences in trajectories and consequently endpoints between two outcome measures both meant to assess dexterity. Our selection of outcome measures, or the state of quantitative outcome measures generally, is one limitation of this study as highlighted by this discrepancy. Interestingly, the more complex mSHAP generated the shorter endpoint with a higher learning rate. This may be due to the greater potential for improvements in strategy and task approach for more complicated tasks, whereas the simpler BBT is more strictly limited to improvements in motor ability.

The learning curve analysis also demonstrated an ability to analyse specific prehensile patterns and task scores within the mSHAP, shedding light on differences in the acquisition of different skills. This could further be used to discuss the specific limitations of certain prostheses or to better focus training based on the progress of individual skills. While designed to address research needs, task and grip specific data (Table 1) may be able to additionally inform clinical rehabilitation by highlighting not only levels of difficulty associated with each task/grip but also presenting expected timelines and trajectories for the acquisition of requisite skills.

Additional findings concerning prepositioning did not support the expected positive correlation with performance. Significantly, the analysis did not demonstrate good fit with the linear model, contradicting not only the expected positive correlation but introducing the possibility of a more nuanced model to explain a potential relationship. Trends from prepositioning data, while inconclusive due to variability, may suggest an optimal amount of prepositioning or a parabolic relationship. With additional work, these findings could be used to inform future training recommendations and ultimately yield another standard aspect of training.

Limited by the longitudinal nature of the study, our smaller sample size likely weakened possible conclusions in the prepositioning analysis. However, in assessing individual scores across training length our sample showed sufficient variability to ensure wide representation. Our reported standard deviation in initial mSHAP scores of 11.50 was larger than the 8.63 standard deviation seen in a similar study employing 47 able-bodied body-powered bypass users [3]. Similarly, a comparison to a 21-subject sample study examining BBT scores showed a standard deviation of 3.7 blocks, comparable to our reported deviation of 4.5 blocks [5].

To gain perspective on the impact of our specific training content, we can again reference data from Huinink (2016) Across the referenced five-session training protocol, Southampton Hand Assessment Procedure scores increased by 15.73 ± 8.08 [3]. Comparatively, across our recommended three-session protocol, mSHAP scores increased 12.95 ± 6.67. While a controlled experiment directly comparing the two training protocols would be needed to eliminate confounds such as procedure modifications and terminal devices used, similarity in our results demonstrates at minimum comparable improvements in performance with fewer sessions and more diverse, representative content. Additionally, existing control data demonstrates an increase of 7.53 ± 6.43 across two Southampton Hand Assessment Procedure administrations, two weeks apart, without any training [3]. Again, these results lend confidence that at a minimum some benefit is seen in our selected training content.

## Conclusion

As a novel training protocol, there are multiple avenues for, and in need of, future work. This protocol could readily be applied and tested similarly for different terminal devices and controls mechanisms (e.g. myoelectric devices) to help generalize its applicability. The effectiveness of the content could be further tested by isolating and varying certain tasks/concepts, such as the direct comparison discussed earlier. Similarly, a comparison to the learning effects seen with repeated mSHAP administration could be performed to distinguish the impact of training in general. This comparison could be further extended to other outcome measures, compared against each other, to distinguish the strengths and weakness of using each measure in this specific context. A final, important, avenue of future work would be a comparison of bypass user performance after training with this protocol, in any number of outcome measures, against amputee users. This would give needed context to the researchers use of bypass users and their unique treatment as research subjects. Ultimately, with the goal of assessing the translatability and validity of using trained bypass users as an analogue for the amputee population.

## Supplementary Material

Supplemental

## Figures and Tables

**Table 1.** Learning curve values for *T*_*s*_ and *LIF*_*pp*_

Scoring Category	Learning Plateau (*a*)	Learning Rate (*b*)	*R*^*2*^	Training endpoint
*Ts*_*lifting a light object*_	81.810	8.036	0.053	1
*Ts*_*rotate key*_	78.153	7.658	0.014	1
*Ts*_*heavy power*_	85.905	17.054	0.739	2
*Ts*_*heavy lateral*_	77.017	13.366	0.063	2
*Ts*_*heavy extension*_	73.555	14.380	0.313	2
*Ts*_*light spherical*_	85.515	16.495	0.407	2
*Ts*_*light lateral*_	80.019	9.191	0.073	2
*Ts*_*heavy spherical*_	77.226	12.991	0.005	2
*Ts*_*heavy tripod*_	68.066	15.068	0.144	3
*Ts*_*heavy tip*_	72.624	20.719	0.185	3
*Ts*_*rotate a screw*_	68.910	13.875	0.040	3
*Ts*_*door handle*_	93.587	22.090	0.940	3
*Ts*_*open/close zip*_	81.115	19.785	0.307	3
*Ts*_*light power*_	89.998	32.045	0.934	4
*Ts*_*heavy object*_	91.678	30.437	0.906	4
*Ts*_*glass jug pouring*_	74.551	25.199	0.589	4
*Ts*_*page turning*_	84.145	28.916	0.574	4
*Ts*_*light tripod*_	77.954	31.493	0.527	5
*Ts*_*light extension*_	78.645	36.015	0.681	5
*Ts*_*carton pouring*_	79.164	34.153	0.602	5
*Ts*_*button board*_	80.999	38.876	0.826	5
*Ts*_*light tip*_	72.735	37.968	0.621	6
*LIF*_*lateral*_	78.171	15.040	0.691	2
*LIF*_*spherical*_	80.635	21.213	0.493	3
*LIF*_*power*_	85.315	20.590	0.823	3
*LIF*_*tripod*_	75.673	28.479	0.818	4
*LIF*_*tip*_	77.125	25.001	0.885	4
*LIF*_*extension*_	78.782	26.437	0.806	4
