# Supplementary material for: Creating a standardized, quantitative training protocol for upper limb bypass prostheses
Source: Phys Med Rehabil Res. Author manuscript; Available in PMC 2019 Jun 4. (PMC6547834)
Supplement: Supplemental [file NIHMS1002758-supplement-Supplemental.pdf]

# Protocol S1

## Protocol

- 1st Session
    - Prosthesis orientation
        - Componentry training
        - Donning and doffing
        - Body control motions
    - Assessment
        - Body control motions, terminal device adjustment, ranges of opening, and use at various heights.
    - Task Training – Blocked order
        - Indirect grasping – 4 trials with each of the three objects resulting in 12 trials. All four trials for an object will be completed before moving on to the next object.
        - Direct grasping – Same structure as indirect grasping
        - Fixation – 4 trials for each of the three tasks resulting in 12 trials. All four trials for a task will be completed before moving on to the next object.
    - Free training
        - 5 minutes sitting and 5 minutes standing
    - mSHAP

- 2nd Session
    - Donning
    - Assessment
        - Body control motions, terminal device adjustment, ranges of opening, and use at various heights
    - Box and blocks test
    - Task training – Random order
        - Indirect grasping, direct grasping, and fixation tasks will be randomized. Only two trials of each task/object combination will be performed.
    - Free Training
        - 5 minutes sitting and 5 minutes standing

- Activities of Daily Living – Blocked order
  - Unilateral ADL tasks will be performed first, followed by the bilateral ADL tasks as time allows within the 20 minutes allocated.
- mSHAP

- 3rd Session
  - Donning
  - Assessment
    - Body control motions, terminal device adjustment, ranges of opening, and use at various heights
  - Box and blocks test
  - Task training – Random order
    - Indirect grasping, direct grasping, and fixation tasks will be randomized. Only two trials of each task/object combination will be performed.
  - Free Training
    - 5 minutes sitting and 5 minutes standing
  - Activities of Daily Living – Random order
    - ADL tasks will be performed in random order.
  - mSHAP

# PROSTHESIS ORIENTATION

## Componentry Training

Introduce the major components of the prosthesis, namely the harness, the control cable, the wrist unit, and the terminal device (TD) while pointing out each on the bypass to the participant. Describe the control cables ability to voluntarily open the TD against passive closure due to the rubber bands. All participants will use the prosthesis with two rubber bands.

**Have the bypass prosthesis, with the control cable detached, on the table.**

> *"The body-powered bypass prosthesis consists of four major components. The harness, which will fit over your shoulders and back, the wrist unit, which will fit over your forearm, the TD which will function in place of your hand, and the control cable, which will connect the harness to the TD such that certain movements will open and close the TD. The rubber band on the TD will then cause passive closure."*
>
> *"Taking a closer look at the TD we note this lever here, often referred to as the thumb, it can be a useful additional point of contact during tasks. Additionally, we can see where the rubber grip extends along the hook, the notch in the grip right at the corner, and how the hooks move and align in relation to one another. Keeping these aspects in mind will help you to use the prosthesis as efficiently as possible."*

*[Point out each component as you discuss. Pull on the control cable to demonstrate voluntary opening of the TD.]*

Demonstrate and discuss the ability to pre-position/rotate the TD by pressing the lever and then pushing the TD to seat it again. Stress the importance of this in maximizing prosthesis functionality.

> *"The TD can be rotated by pressing this lever to release and then pushing the TD in to seat again, indicated by the click you hear. This feature is important to consider due to the inability to rotate the wrist unit. Therefore, prior to starting a task, time should be taken to think through the necessary grip and to rotate the TD accordingly."*

*[Demonstrate the ability to rotate and seat the TD.]*

## DONNING AND DOFFING

Help the participant into the prosthesis while describing each step such that the participant can don the prosthesis without assistance in the future. The control cable should be disconnected during donning and doffing to prevent damaging the prosthesis.

**Adjust the harness straps preemptively erring on the side of too tight based on the size of the participant (it is simpler to loosen the straps than to tighten).**

> *"Place your right hand through the anterior support strap, through the wrist unit, and grasp the handle. Tighten the straps on your forearm. Take your other hand and insert through the axilla loop."*

*[Assist the participant donning the prosthesis. In order adjust the axilla loop tension, the anterior support strap, y straps, and then the control strap. Continually engage the participant ensuring the fit is comfortable and secure. The ring harness should be inferior and to the sound side of the vertebra prominens; the y-strap should rest at the axilla.]*

## BODY CONTROL MOTIONS

The prosthesis is controlled through two main body control motions, shoulder flexion/extension and scapular protraction. Describe and demonstrate each motion and have the participant mirror your motions. Stress the importance of thinking and selecting from these control motions prior to performing a task.

> *"The TD can be opened through specific body control motions. Shoulder flexion/extension and scapular protraction, which can be performed in 3 ways: Bypass side, sound side, or biscapular. It is important to think through these control motions and select a strategy before performing a task."*

*[Demonstrate each movement as you discuss it and have the participant mirror your motions.]*

Give the participant additional time to try each motion on their own and offer to make any adjustments to the harness now.

## 1ST ASSESSMENT

A general assessment of prosthesis control, prior to task training, will be performed to ensure participants have an adequate understanding of the prosthesis in order to communicate effectively and to use the prosthesis safely.

> *"Before moving on to work with actual objects and grasping we need to go through a quick assessment to ensure you understand how to properly use the prosthesis."*

Have the participant demonstrate body control motions, adjustment and seating of the TD, various ranges of TD opening and closing, and various heights of wrist unit during opening and closing. This assessment is not designed to be restrictive, if the participant answers incorrectly prompt them with an explanation and quick demonstration and ask them to repeat.

> *"Open and close the TD using shoulder flexion/extension."*
>
> *"Open and close the TD using bypass side scapular protraction"*
>
> *"Open and close the TD using sound side scapular protraction."*
>
> *"Open and close the TD using biscapular protraction."*
>
> *"Adjust the TD orientation and reseat it."*
>
> *"Using any body control motion you like open the TD fully… open the TD half-way."*
>
> *"Using any body control motion you like open the TD at waist level… at chest level… at face level."*

*[Engage participant and adjust any straps as needed. Observe when the TD begins opening through the body control motion and ensure harness fit allows proper activation.]*

Participants must demonstrate mastery of these skills prior to moving on to task training. Continue coaching and repeating this assessment as necessary until the participant completes it satisfactorily. Again, offer to make any adjustments to the prosthesis now.

## BOX AND BLOCKS TEST

The Box and Block Test (BBT) measures unilateral gross manual dexterity and involves transporting as many blocks as possible from one side of the box, over a partition, to the other side in a minute. The BBT should be administered immediately following the initial assessment in each session, excluding the 1st session.

**The test box is placed lengthwise along a line 5 inches from the edge of the table. The participant should be seated with the table at a height that allows their hands to rest on top of the box with their bypass side elbow at 90 degrees and their hands on either side of the box. 150 blocks should be in the right compartment. The examiner should face the participant to view the test.**

> *"I want to see how quickly you can pick up one block at a time with the prosthesis [point to prosthesis], carry it to the other side of the box, and drop it. Make sure the TD crosses the partition. Watch me while I show you how."*

*[Transport three cubes over the partition in the same direction you want the participant to move them.]*

> *"If you pick up two blocks at a time, they will count as one. If you drop one on the floor or table after you have carried it across, it will still be counted, so do not waste time picking it up. If you toss the blocks without your fingertips crossing the partition, they will not be counted. Before you start, you will have a chance to practice for 15 seconds. Do you have any questions?"*
>
> *"Then place your hands on the sides of the box. When it is time to start, I will say ready and then go."*
>
> *"Ready…Go…Stop"*

*[Start the watch at the word go. When 15 seconds has passed, say "stop." Inform the participant of any mistakes they may have made during the practice period. Reset the test.]*

> *"This will be the actual test. The instructions are the same. Work as quickly as you can."*
>
> *"Ready…Go…Stop"*

*[Start the watch at the word go. When 1 minute has passed, say "stop." Remove blocks that weren't properly transported. Count and record the number of blocks. Reset the test.]*

The test should be repeated when the participant is ready for a total of 3 trials.

## TASK TRAINING

### OVERVIEW

The participant will perform 3 forms of basic object manipulation tasks. Indirect grasping (IG) and direct grasping (DG) tasks will follow the same procedure for each trial but will utilize 3 different objects; a wooden block, a foam ball, and a metal can. Fixation tasks will involve 3 unique tasks. These tasks will be performed seated.

In blocked order sessions all 4 trials for an object/task will be completed before moving on to the next object/task. In the random order sessions objects/tasks will be randomized and only 2 trials will be performed for each object/task combination.

Tasks will be performed on a table top with standardized dimensions and site locations for hands and objects. The template is designed to cover a 122 x 77 cm table top. The far horizontal line (the mSHAP line) marks 20 cm from the near edge of the table; the vertical line marks the midline of the table. Each circle is 10 cm in diameter. The left and right circles' centers are both 40 cm from the near edge of the table and 20 cm from midline. The mid circle's center is 25 cm from the near edge of the table. The squares (home) are aligned vertically with the left and right circles, are centered 10 cm from the near edge of the table and are 10 x 10 cm. The short horizontal line between the home squares (the BBT line) is 5 inches from the near edge of the table.

Outlines of the 3 x 3 cm block and 8 cm diameter can used in training can be found in the mid and right circles. These can be used as an additional challenge for participants by prompting them to release the object within the shape in either grasping task.

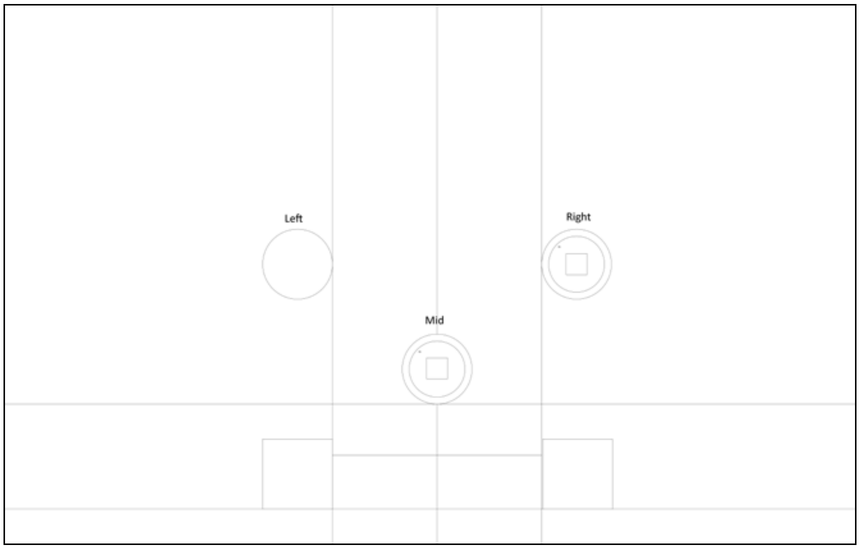


*The table template is used to guide the tasks. Locations labelled on the template are referred to as right circle, left circle, and mid circle in the task instructions. Midline refers to the vertical line centered in the template. Home refers to the 2 squares. The mSHAP line refers to the far horizontal line. The BBT line refers to the horizontal line between the home squares.*

**Have the wooden block, foam ball, and metal can nearby. The table should be clear except for the template. The participant should be seated with their midline aligned with the vertical line on the table template.**

> *"Now we'll move on to object manipulation tasks. Each grasping task will be performed 4 times each for 3 different objects. The grasping tasks are either IG, in which the object is placed into the TD with the free hand, or direct grasping, in which the object is picked up directly with the TD. Each of the 3 fixation tasks will be completed 4 times. The fixation tasks involve completing a task with your free hand while using the TD to stabilize, or fixate, the object. These tasks will not be timed and are for training purposes only, therefore we encourage you to take your time and discuss each task."*

For the 1st trial of each object/task prompt the participant to explain the grip strategy and body control motion that they plan to use prior to starting the task.

> *"Before beginning the task I want you to plan your strategy and explain it to me. Be sure to include TD orientation, how the object will be situated in the grip, and body control motions for both grasp and release."*

Before the 2$^{nd}$ trial, prompt the participant to describe any difficulties they found during the 1st trial and any changes they plan to make in their second attempt.

> *"What difficulties did you find with the task and with your particular strategy? How do you plan on performing the task differently this time?"*

Before the 3$^{rd}$ trial offer any coaching on strategy if appropriate. Finally, before and after the 4$^{th}$ trial offer feedback on what worked in their strategies and what can be improved.

*[Prompts for the 3rd and 4th trials will be based on the participants' unique performance and can't be standardized. Suggestions commonly focus on TD orientation, isolation of body control motions during tasks, use of compensatory movements, etc.]*

## INDIRECT GRASPING

IG tasks involve placing an object into the grip of the TD using the free hand. The object will begin in the left circle, be grasped by the left hand, transferred to the TD in midair at the midline, and be released in the right circle. Both hands will start at home.

**Place one of the objects in the left circle.**

> *"First we'll do the IG tasks. A series of objects will be placed on the table in front of you in the left circle and you will move them to the right circle as labeled on the table. In this task your hands will start at home, you will grasp the object in your left hand from the left circle. You will transfer the object to your TD at the midline in*

> *midair. You will then place that object in the right circle and release it using only the TD."*

*[Demonstrate the procedure yourself while explaining the task to the participant. Deliver the appropriate prompt for each trial.]*

## DIRECT GRASPING

DG tasks involve picking up and moving an object solely with the TD. The object will begin in the right circle, be grasped by the TD, and released in the mid circle. Both hands will start at home.

**Place one of the objects in the right circle.**

> *"Now we'll move to DG tasks. A series of objects will be placed on the table in front of you in the right circle and you will move them to the mid circle as labeled on the table. In this task your hands will start at home, you will grasp the object with your TD from the right circle. You will then place that object in the mid circle and release it using only the TD."*

*[Demonstrate the procedure yourself while explaining the task to the participant. Reset the object following your demonstration and after each trial. Deliver the appropriate prompt for each trial]*

## FIXATION

FIX tasks involves performing a task with the free hand while the TD acts as a stabilizer. The objects are centered over the mid circle.

### *RULER TASK*

Participants will fixate (or hold steady) a ruler between two dots drawn on a piece of paper 20 cm apart with the TD. Using their free hand and the ruler they'll draw a straight line between the two dots. The participant can use their free hand to move the ruler but must use the prosthesis to fixate the ruler while drawing. Both hands will begin at home.

**The piece of paper should be centered over the mid circle with the ruler situated along the two dots drawn 20 cm apart. The pen should start in the left circle.**

> *"Now we'll move to FIX tasks. In this task you will use the TD to fixate, or stabilize, the ruler between the two dots on the paper in front of you. You will use your left hand to draw a straight line between the dots. Your hands will start at home, you will grasp the pen from the left circle, you will draw a straight line between two dots*

> *using the ruler, you will fixate the ruler with the TD, and then you will release the pen in the left circle."*

*[Demonstrate the procedure yourself while explaining the task to the participant. Reset the object following your demonstration and after each trial. Deliver the appropriate prompt for each trial]*

### *ZIPPER TASK*

In the second task participants will fixate a piece of cloth with a zipper using the TD. Using their left hand, they'll open and close the zipper. Both hands will begin at home.

**The zipper should be centered over the mid circle. The zipper should be fully zipped.**

> *"In this task you will use the TD to fixate the cloth in front of you. You will use your left hand to unzip the zipper fully and then zip the zipper closed."*

*[Demonstrate the procedure yourself while explaining the task to the participant. Deliver the appropriate prompt for each trial]*

### *BUTTON TASK*

In the third task the participant will fixate a piece of cloth with four buttons with the TD. Using the free hand, they'll unbutton each button. Both hands will begin at home.

**The button board should be centered at the mid site with the buttons aligned horizontally.**

> *"In this task you will use the TD to fixate the board in front of you. You will use your left hand to grasp the cloth and unbutton each button. "*

*[Demonstrate the procedure yourself while explaining the task to the participant. Deliver the appropriate prompt for each trial]*

## FREE TRAINING

Allow the participant 12 minutes to use the prosthesis as they wish. Encourage them to interact with the objects laid out on the table. Participants will remain seated, with the table at the sitting height, for 5 minutes and then will stand, with the table at the standing height, for the remaining 5 minutes.

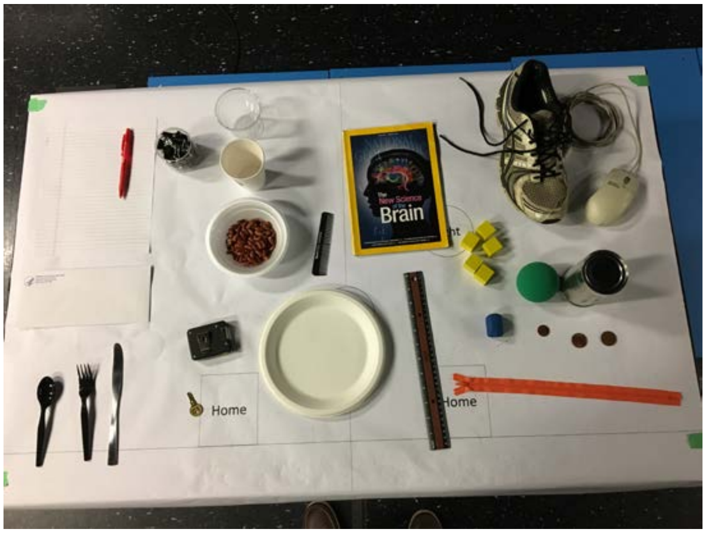


Table full of free training materials for use by the participant

## ACTIVITIES OF DAILY LIVING

### OVERVIEW

12 ADLs have been chosen to provide additional training. Participants should be encouraged to discuss strategies with the research assistant. Each ADL will be performed twice. In the blocked order sessions, the unilateral tasks and then the bilateral tasks will be performed in the order they appear below. In the random order sessions, the task trials will be randomized. ADL training will last for 20 minutes, independent of the number of tasks performed within that time, to give the participant and the research assistant flexibility in training. Only the prosthesis should be used in unilateral tasks unless otherwise stated. The prosthesis and free hand can both be used in bilateral tasks.

**The table should be cleared except for the table template. The ADL materials should be nearby and ready for use.**

> *"Now we're going to work through some activities of daily living, or ADLs, these tasks are based on real-life tasks and require combinations of movements and more complex strategies. We encourage you to discuss movement strategies with us prior to starting a task and to actively think through your movements."*

For the 1st trial of each object/task prompt the participant to explain the grip strategy and body control motion that they plan to use prior to starting the task.

> *"Before beginning the task I want you to plan your strategy and explain it to me. Be sure to include TD orientation, how the object will be situated in the grip, and body control motions for both grasp and release."*

Before the 2$^{nd}$ trial, prompt the participant to describe any difficulties they found during the 1st trial and any changes they plan to make in their second attempt.

> *"What difficulties did you find with the task and with your particular strategy? How do you plan on performing the task differently this time?"*

Before moving on to the next task give feedback on their strategy, what worked and what didn't, and anything they may have done differently.

*[Feedback following the 2nd trial will be based on the participants' unique performance and can't be standardized. Suggestions commonly focus on TD orientation, isolation of body control motions during tasks, use of compensatory movements, etc.]*

### UNILATERAL TASKS

#### *BRUSH HAIR*

Instruct the participant to grasp the brush, bring the brush to their head, brush their hair or perform a brushing motion, and release the brush from grasp.

**The comb should be aligned horizontally and centered in the mid circle. The participant should be standing with both hands at home.**

> *"Please take this brush and, while grasping it, run it through your hair (or over the top of their head if bald). Then put the brush down in the mid circle. Try to use only your prosthesis to do this task."*

*[Demonstrate the procedure yourself while explaining the task to the participant. Deliver the appropriate prompt for each trial. Reset the task.]*

### *DRINK FROM A CUP*

Instruct the participant to pick up the cup from the table, bring the cup to their mouth, tilt the cup and simulate drinking, return the cup to the table in the mid circle, and release their grip on the cup.

**The cup should be centered in the mid circle. The participant should be standing with both hands at home.**

> *"Pick up this paper cup from the table, bring it all the way to your mouth, and pretend to drink from it. Then put the cup back on the table in the mid circle. Try to use only your prosthesis to do this task."*

*[Demonstrate the procedure yourself while explaining the task to the participant. Deliver the appropriate prompt for each trial. Reset the task.]*

### *USE A FORK*

Instruct the participant to grasp the fork, use the fork to stab the plasticine, bring the fork and plasticine all the way to the mouth as if taking a bite of food, move the fork away from their mouth and return it to the mid circle, and release their grip on the fork.

**The fork should be aligned vertically and centered in the mid circle. The plasticine should be shaped into a ball and centered in the left circle. The participant should be sitting with both hands at home.**

> *"Please grasp this fork, stab the plasticine, and bring it to your mouth as if you were going to take a bite of food from it. Then put the fork back on the table in the mid circle. Try to use only your prosthesis to do this task"*

*[Demonstrate the procedure yourself while explaining the task to the participant. Deliver the appropriate prompt for each trial. Reset the task.]*

### *USE A SPOON*

Instruct the participant to grasp the spoon, use the spoon to scoop bean(s) from the bowl, bring the spoon and bean(s) all the way to their mouth as if taking a bite of food, move the spoon away from their mouth, deposit the bean(s) in the bowl, return the spoon to the mid circle, and release their grip on the spoon.

**The spoon should be aligned vertically and centered in the mid circle. The bowl of beans should be centered in the right circle. The participant should be sitting with both hands at home.**

> *"Please grasp this spoon with your prosthesis, use it to scoop a bean(s) from the bowl, and bring the spoon and bean(s) to your mouth as if you were going to take a bite from it. Then put the bean(s) back in the bowl and the spoon back on the table in the mid circle. Be sure to use your prosthesis while doing this."*

*[Demonstrate the procedure yourself while explaining the task to the participant. Deliver the appropriate prompt for each trial. Reset the task.]*

### *POUR FROM A CAN*

Instruct the participant to pick up the can, pour the contents into the cup, set the can down on the table, and release the can.

**The can should be filled with 100 mL of water and centered in the mid circle, with the tab open and pointing to the far edge of the table. The cup should be centered in the left circle. The participant should be sitting with both hands at home.**

> *"Please pick up the soda can from the table. Then pour the water from the can into the cup, making sure not to spill any water. When finished set the can down on the table upright in the mid circle. Be sure to use your prosthesis."*

*[Demonstrate the procedure yourself while explaining the task to the participant. Deliver the appropriate prompt for each trial. Reset the task.]*

### *WRITE LEGIBLY*

Instruct the participant to grasp the pen, write the word "LETTER", set the pen down on the table in the mid circle, and release their grip

**The paper should be aligned vertically, centered over the mid circle. The pen should be aligned vertically with the tip towards the participant, centered over the right circle. The pen should be 'clicked' close. The participant should be sitting with both hands at home.**

> *"Please grasp this pen with your prosthesis, click it open, and write the word "LETTER" on the blank sheet of paper on the table. Then set the pen down on the table in the right circle. Try to use only your prosthesis to do this task. You may use your left hand to stabilize the paper"*

*[Demonstrate the procedure yourself while explaining the task to the participant. Deliver the appropriate prompt for each trial. Reset the task.]*

## BILATERAL TASKS

### *ZIP VEST*

Instruct the participant to don the vest, initiate the zipper, pull the zipper up at least two thirds of the way, and then unzip the zipper.

**The vest should be laid with the collar towards the far edge of the table, unzipped with the front facing upwards, centered over the mid circle. The participant should be standing with both hands at home.**

> *"Please put this vest on (therapist may help don vest), start the zipper, and pull it at least two-thirds of the way up. Then unzip the zipper. Be sure to use your prosthesis while doing this."*

*[Demonstrate the procedure yourself while explaining the task to the participant. Deliver the appropriate prompt for each trial. Reset the task.]*

### *TIE SHOELACES*

Instruct the participant to grasp a lace with each hand, crisscross the laces, make loops, pull the bow tight, and release their grasp on the laces.

**The shoe should be positioned with the toe closest to the far edge of the table, centered over the mid circle. The participant should be standing with both hands at home.**

> *"Please take this shoe and tie the laces. Grasp a lace in each hand, crisscross the laces, making loops, and tie the bow tight. Be sure to use your prosthesis while doing this."*

*[Demonstrate the procedure yourself while explaining the task to the participant. Deliver the appropriate prompt for each trial. Reset the task.]*

### *FOLD A BATH TOWEL*

Instruct the participant to grasp the ends of the towel, lift the towel off the table, bring the ends together to fold twice in two different directions, and release their grasp on the towel.

**The towel should be centered over the mid circle. The participant should be standing with both hands at home.**

> *"Please grasp the ends of this bath towel and hold the towel off the table, then bring the ends of the towel together to fold it twice in two different directions. Place the folded towel back on the table. Be sure to use your prosthesis while doing this."*

*[Demonstrate the procedure yourself while explaining the task to the participant. Deliver the appropriate prompt for each trial. Reset the task.]*

### *OPEN AN ENVELOPE*

Instruct the participant to grasp the envelope in the TD, use a pen break the top seal of the envelope, open the envelope, remove the paper within the envelope, place the envelope and contents over the mid circle and the pen in the left circle, and release the envelope, its contents, and the pen.

**The envelope should be centered over the mid circle. The pen should be oriented vertically, tip pointing down, centered in the left circle. The participant should be sitting with both hands at home.**

> *"Please secure this envelope and, using a pen, break the seal on it. Then open the envelope, remove it contents, and place both on the table over the mid circle. Return the pen to the left circle. Be sure to use your prosthesis while doing this."*

*[Demonstrate the procedure yourself while explaining the task to the participant. Deliver the appropriate prompt for each trial. Reset the task.]*

### *TAPE MEASURE*

Instruct the participant to grasp the tape measure, extend the tape to measure between the mid circle and the right circle, withdraw the tape, place the tape measure over the mid circle, and release their grip.

**The tape measure should be centered in the left circle, with the tape withdrawn and oriented on the right. The participant should be sitting with both hands at home.**

> *"Please grasp the tape measure and measure the distance between the boundaries of the mid circle and the right circle. Then withdraw the tape and place it in the mid circle. Be sure to use your prosthesis while doing this."*

*[Demonstrate the procedure yourself while explaining the task to the participant. Deliver the appropriate prompt for each trial. Reset the task.]*

### *OPEN A BAG*

Instruct the participant to grasp the Ziploc bag, open the bag in the air (without fixating against the table), grasp the pen, place the pen in the Ziploc bag, seal the bag, place the bag in the mid circle, and release their grip.

**The Ziploc bag should be centered over the mid circle. The pen should be aligned horizontally, centered in the left circle. The participant should be standing with both hands at home.**

> *"Please grasp the Ziploc bag and, holding it off the table, grasp the pen and place it in the bag. Then seal the bag and place it back on the table over the mid circle. Be sure to use your prosthesis while doing this."*

*[Demonstrate the procedure yourself while explaining the task to the participant. Deliver the appropriate prompt for each trial. Reset the task. Don't allow the participant to fixate the bag or pen against their body.]*

## MODIFIED SOUTHAMPTON HAND ASSESSMENT PROCEDURE

### OVERVIEW

The Southampton Hand Assessment Procedure (SHAP) is based on the analysis of grip patterns, and their frequency of use in Activities of Daily Living (ADL) tasks. In this modified version changes have been made to accommodate body-powered bypass prosthesis use and to simplify the process of scoring.

The participant should be seated with the table at the appropriate height. For each of the tasks the board should be moved from left to right so that each task is directly in front of the participant's prosthesis. Each task should be demonstrated to the participant using slow and clear movements.

Participants can practice each task for a maximum of 1 minute, prior to timing it, to determine the most appropriate technique. Only a single attempt will be allowed for each task, excluding errors associated with the timer. Incomplete attempts should be recorded as "c/c" (could not complete); these include significant spills, an object falling from the board or case, an incorrect final position (ie. the object is not in the slot), and use of any body part other than the TD unless otherwise specified. Participants should be instructed to continue the task to completion to avoid "c/c" even in the event of errors. During practice the administrator should point out any attempts that would be considered incomplete to ensure errors are associated only with execution and not understanding.

In the appropriate forms (See Appendix) the time and any additional notes should be recorded. Additionally, the quadrant choice in the bypass assessments should be recorded. The quadrant choice is a means to identify the orientation of the TD defined by the direction of the hook relative to the wrist unit.

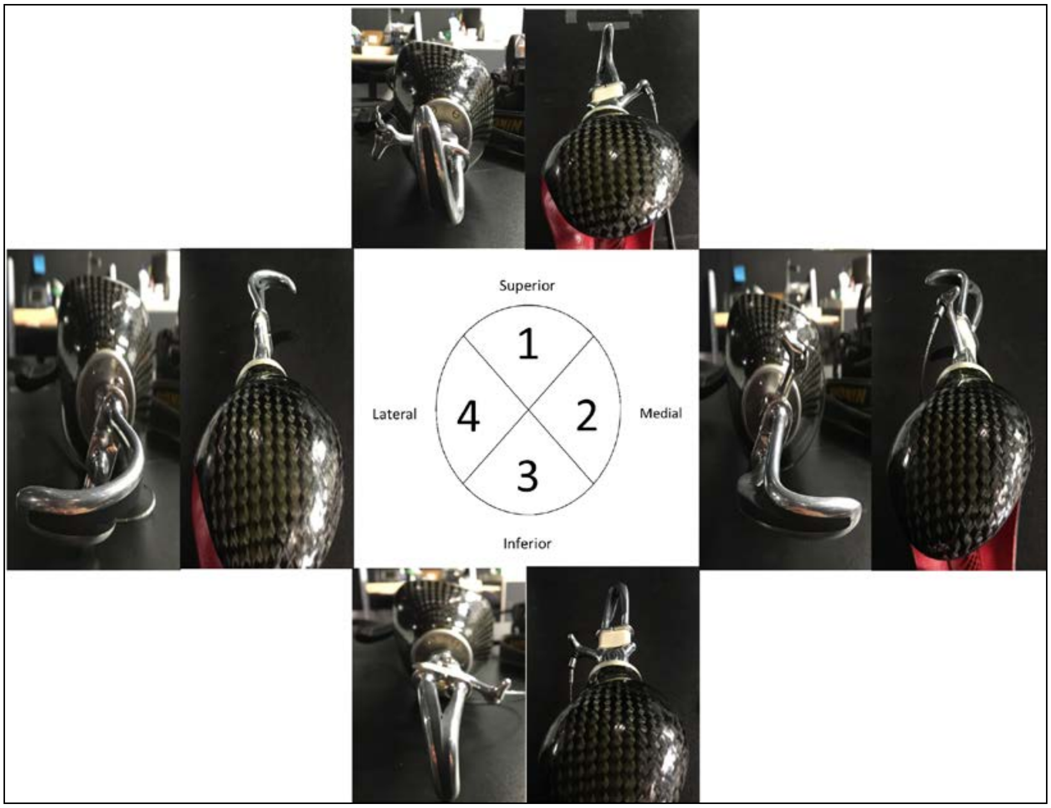

*The direction that the end of the hook points relative to the wrist unit falls into one of four different quadrants when recording the orientation of the TD.*

The mSHAP is divided into abstract objects, heavyweight and lightweight, and ADLs, board and case. The abstract objects task should be randomized, including both heavyweight and lightweight versions, and performed first. The ADL tasks should be randomized only within their respective groups of board or case and performed second. Which group begins within the ADL portion, board or case, should additionally be randomized.

> *"Now we'll conduct a modified version of the Southampton Hand Assessment Procedure or mSHAP to establish your functionality with the bypass prosthesis. This procedure consists of a series of grasping tasks with 12 different abstract objects and a series of 10 activities of daily living, or ADLS. Your functionality will be determined by how quickly you complete each task. 1 minute of practice will be allowed prior to each task to work through different strategies on your own. Only a single attempt will be allowed following practice, excluding any errors associated with the timer. Any task considered incomplete following the stopping of the timer will be recorded as "could not complete" resulting in the maximum allowed time, therefore do not stop the timer following an error until a task is completed or until I tell you to stop. I can additionally confirm correct completion of a task during the practice time."*

## ABSTRACT OBJECTS

Both lightweight and heavyweight versions follow the same procedure; the lightweight objects are the wooden versions, while the heavyweight objects are metal. Each object will be moved from its corresponding rear slot to the front slot using the right hand.

**Place the SHAP board on the table blue side up. Align the front edge of the board with the mSHAP line on the table template. Have all the abstract objects out of the case and organized on a table nearby. Have the participant sit at the table with their midline aligned with the vertical line on the table template.**

> *"A series of objects will be placed on the board. The task involves moving the object from the rear slot on the board to the front slot. Only the hand under assessment (dominant hand) should be used for any of these tasks, including the starting and stopping of the timer."*

*[Point out the rear slot and the front slot for an abstract object]*

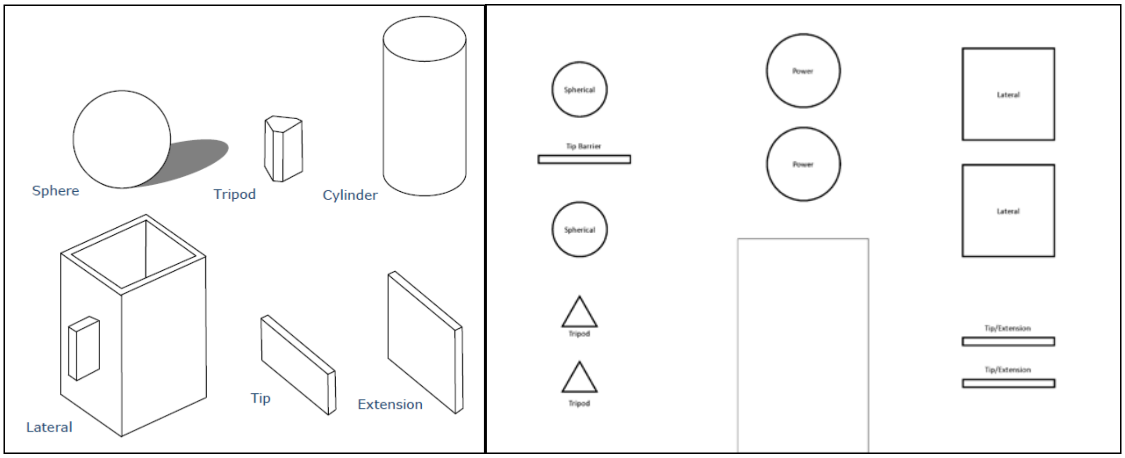


*Diagram of the abstract objects (left) and the abstract objects side of the SHAP board labeled for each object (right). The object labeled cylinder corresponds to the power grip tasks.*

### *SPHERICAL*

**Place the 'spherical object' in its rear slot. Place the 'heavyweight tip object' in the slot between the rear and front 'spherical object' slots to create a small barrier. Align the object with the participant's dominant hand.**

> *"Start the timer, pick up and move the object as demonstrated with as few mistakes as possible, and as quickly as possible, to the front slot. Complete the task by depressing the blue button on the timer again."*

*[Use the spherical grip to move the object over the barrier and place it in the front slot and reset the task afterwards. Start timer for 1 minute to enforce practice time limit. Record the participant's time.]*

### *TRIPOD*

**Place the 'tripod object' in the appropriate rear slot. Align the object with the participant's dominant hand.**

> *"Start the timer, move the object as demonstrated and as quickly as possible to the front slot and then stop the timer."*

*[Use a tripod grip to move the object to the front slot and reset the task afterwards. Start timer for 1 minute to enforce practice time limit. Record the participant's time.]*

### *POWER*

**Place the 'power object' in the appropriate rear slot. Align the object with the participant's dominant hand**

> *"Start the timer, move the object as demonstrated and as quickly as possible to the front slot and then stop the timer."*

*[Use a power grip to move the object to the front slot and reset the task afterwards. Start timer for 1 minute to enforce practice time limit. Record the participant's time.]*

### *LATERAL*

**Place the 'lateral object' in the appropriate rear slot with the handle facing toward the participant. Align the object with the participant's dominant hand**

> *"Start the timer, move the object as demonstrated and as quickly as possible to the front slot and then stop the timer."*

*[Use a lateral grip to move the object to the front slot, using the handle, and reset the task afterwards. Start timer for 1 minute to enforce practice time limit. Record the participant's time.]*

### *TIP*

**Place the 'tip object' in the appropriate rear slot. Align the object with the participant's dominant hand.**

> *"Start the timer, move the object as demonstrated and as quickly as possible to the front slot and then stop the timer."*

*[Use a tip grip to move the object to the front slot and reset the task afterwards. Start timer for 1 minute to enforce practice time limit. Record the participant's time.]*

### *EXTENSION*

**Place the 'extension object' in the appropriate rear slot. Align the object with the participant's dominant hand.**

> *"Start the timer, move the object as demonstrated and as quickly as possible to the front slot and then stop the timer."*

*[Use an extension grip to move the object to the front slot and reset the task afterwards. Start timer for 1 minute to enforce practice time limit. Record the participant's time.]*

## ACTIVITIES OF DAILY LIVING

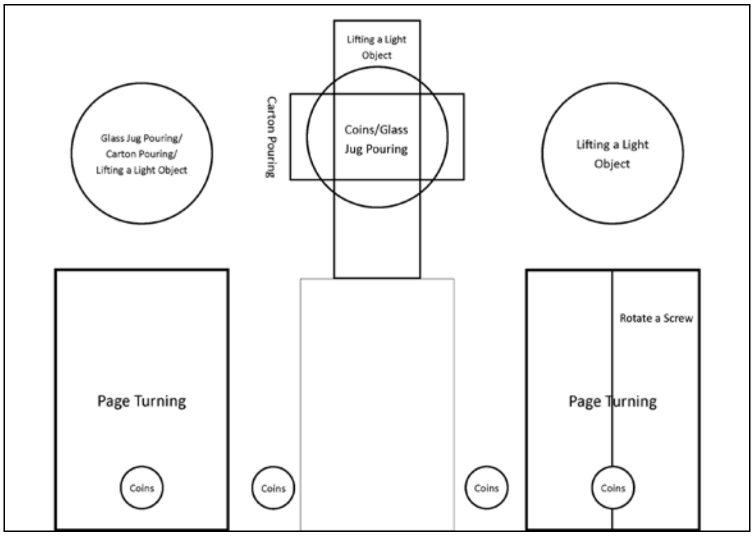


*The ADL side of the SHAP board labeled for each task and the locations of the corresponding objects.*

**Place the SHAP board on the table white side up. Align the front edge of the board with the mSHAP line on the table template. Have all the ADL objects out of the case and organized on a table nearby. Have the participant sit at the table with their midline aligned with the vertical line on the table template. Fill the jar to the top of the label with water for use in 'Moving a heavy object', 'Jug pouring', and 'Carton pouring' tasks.**

> *"The second stage of this assessment consists of 10 everyday activities, which should be timed in the same manner by pressing the blue button to start and stop the timer. Again, tasks should be completed as quickly as possible, with as few mistakes as possible, using only the appropriate hand unless otherwise stated."*

## *Board tasks*

### *CARTON POURING – SPHERICAL (CARTON)*

**Fill the carton with 200ml of water. Place the glass jar (without the lid) on the designated left area. Place the carton in the designated area on the SHAP board with the spout of the carton pointing toward to glass jar. Ensure the timer is in its slot on the SHAP board and the objects starting position is aligned with the participant's dominant hand.**

> *"Start the timer and whilst ensuring as little spillage as possible, pour the water from the carton into the jar as demonstrated and as quickly as possible. Replace the carton on the board and then stop the timer."*

*[Pick up the carton using a spherical grip and show how to pour the water into the glass jar then reset the task for the participant. Start timer for 1 minute to enforce practice time limit. Record the participant's time.]*

***BUTTON BOARD – TRIPOD (BUTTON)***

**Place the button board to the right of the timer unit. The buttons should be farthest from the timer unit. Ensure the timer is in its slot on the SHAP board and the objects starting position is aligned with the participant's dominant hand.**

> *"Start the timer and using only the appropriate hand, undo all four buttons in any order as demonstrated and as quickly as possible. You may steady the button board with your other hand so that it remains on the SHAP board throughout the task. Then stop the timer using only the appropriate hand."*

*[Undo each button in turn, using only the assessed hand in a tripod grip and reset the task for the participant. Start timer for 1 minute to enforce practice time limit. Record the participant's time.]*

***LIFTING A HEAVY OBJECT – POWER (HEAVY)***

**Fill the glass jar with water to the top of the label and tighten the lid. Place the jar in the designated left area on the SHAP board. Place the empty carton lengthways along the middle of the SHAP board (without obstructing the timer unit) to create a barrier. Ensure the timer is in its slot on the SHAP board and the objects starting position is aligned with the participant's dominant hand.**

> *"Start the timer, move the jar over the carton to the other side of the board as demonstrated and as quickly as possible. Then stop the timer."*

*[Lift the jar over the carton using a power grip and place on the opposite side of the form board in the designated area. Reset the task for the participant. Start timer for 1 minute to enforce practice time limit. Record the participant's time.]*

***LIFTING A LIGHT OBJECT – POWER (LIGHT)***

**Place the empty tin (with the plastic lid on) in the designated left area. Place the carton on its side in its designated area as a barrier. Ensure the timer is in its slot on the SHAP board and the objects starting position is aligned with the participant's dominant hand.**

> *"Start the timer, move the tin over the carton to the other side of the board as demonstrated and as quickly as possible. Then stop the timer."*

*[Lift the tin over the carton using the power grip and place on the opposite side of the SHAP board in the designated area, then reset the task for the participant. Start timer for 1 minute to enforce practice time limit. Record the participant's time.]*

### *GLASS JUG POURING – LATERAL (JUG)*

**Fill the glass jug with 100ml of water. Place the jug in the designated area of the SHAP board with the handle of the glass jug pointing the right. Place the glass jar (without the lid) on the designated left area. Ensure the timer is in its slot on the SHAP board and the objects starting position is aligned with the participant's dominant hand.**

> *"Start the timer and whilst ensuring as little spillage as possible, pour the water from the jug into the jar as demonstrated and as quickly as possible. Replace the jug on the board and then stop the timer."*

*[Lift the glass jug by the handle using a lateral grip and show how to pour the water into the glass jar. Reset the task for the participant. Start timer for 1 minute to enforce practice time limit. Record the participant's time.]*

### *PAGE TURNING – EXTENSION (PAGE)*

**Place the piece of card in the designated area on the opposite side of the platform to the hand under assessment. Ensure the timer is in its slot on the SHAP board.**

> *"Start the timer lift and turn over the card as if you were turning the pages of a book and place the card on the opposite side of the board as demonstrated and as quickly as possible. Then stop the timer."*

*[Using an extension grip, pick up the card, turn it over as if turning the pages of a book and place it on the opposite side of the SHAP board. Reset the task for the participant. Start timer for 1 minute to enforce practice time limit. Record the participant's time.]*

## *Case tasks*

### *ROTATE A SCREW – POWER (SCREW)*

**Place the screwdriver in the designated area on the board on the right side. The arrow unit is mounted on a clip, which should be attached to the front of the case. Use the area directly in front of the screwdriver between the lock and the handle on the case. Ensure the arrow is pointing upward. Ensure the timer is in its slot on the SHAP board and the objects starting position is aligned with the participant's dominant hand.**

> *"Start the timer and use the screwdriver to rotate the screw a quarter turn clockwise to, or beyond the white mark as demonstrated and as quickly as possible. Once completed, the screwdriver should be replaced on the platform and the timer stopped. Two hands may be used to guide the screwdriver to the screw, but only the appropriate hand should be used for turning the screw. Your other hand can be used to steady the top of the arrow unit."*

*[Demonstrate the task using a power grip. Reset the task for the participant. Start timer for 1 minute to enforce practice time limit. Record the participant's time.]*

***DOOR HANDLE – POWER (HANDLE)***

**Ensure the timer is in its slot on the SHAP board and the objects starting position is aligned with the participant's dominant hand.**

> *"Start the timer, rotate the door handle until it is fully open and then release it as demonstrated and as quickly as possible. Then stop the timer."*

*[Rotate the door handle using a power grip until it is fully open, and then release the handle. Reset the task for the participant. Start timer for 1 minute to enforce practice time limit. Record the participant's time.]*

***ROTATE KEY – TIP (KEY)***

**Place the board in the case ADL side up on top of the foam insert (the timer unit should fit neatly in its original position on the board without moving it from the foam). Ensure the key is aligned with the participant's dominant hand.**

> *"Start the timer, rotate the key as demonstrated and as quickly as possible to the white mark and release the key (at which time the key will spring back to its start position) and then stop the timer."*

*[Turn the key to the white mark using the tip grip. Reset the task for the participant. Start timer for 1 minute to enforce practice time limit. Record the participant's time.]*

***OPEN/CLOSE ZIP – LATERAL/TIP (ZIP)***

**Ensure the zip is closed. Ensure the timer is in its slot on the SHAP board and the objects starting position is aligned with the participant's dominant hand.**

> *"Start the timer, open and close the zip as demonstrated and as quickly as possible and then stop the timer."*

*[Open and close the zip using a lateral or tip grip. Start timer for 1 minute to enforce practice time limit. Record the participant's time.]*

**Table S1 - mSHAP Results Subject 1**

Succesful completion times (s) for each task of the mSHAP for each session for Subject 1. DNF recorded for unsuccessful attempts

| Tasks | Session 1 | Session 2 | Session 3 | Session 4 | Session 5 | Session 6 | Session 7 | Session 8 | Session 9 | Session 10 |
|---|---|---|---|---|---|---|---|---|---|---|
| **Light Spherical** | 3.87 | 4.28 | 5.19 | 4.81 | 4.38 | 5.03 | 3.28 | 2.63 | 3.22 | 2.47 |
| **Light Tripod** | 14.03 | 6.03 | 5.35 | 6.34 | 4.97 | 4.32 | 3.15 | 4.8 | 3.22 | 3.19 |
| **Light Power** | 4.07 | 5.16 | 5.12 | 5.28 | 5 | 3.65 | 3.65 | 2.69 | 2.78 | 2.84 |
| **Light Lateral** | 4.47 | 5.75 | 4.68 | 4.63 | 4.66 | 4.57 | 7.32 | DNF | 2.81 | 2.59 |
| **Light Tip** | 14.34 | 8 | 9.71 | 8.84 | 6.07 | 7.18 | 4.62 | 3.38 | 5.12 | 3.38 |
| **Light Extension** | 6.15 | 8.82 | 7.65 | 7.15 | 5.12 | 7.28 | 4.81 | 4.16 | 3.37 | 3.69 |
| **Heavy Spherical** | 4.88 | 7.34 | 4.12 | DNF | 5.62 | 5.5 | 3.31 | 4.03 | 3.16 | 2.66 |
| **Heavy Tripod** | 8.87 | 5.84 | 5.22 | 5.88 | 4.63 | 5.31 | 3.57 | 21.75 | 4.16 | 6.31 |
| **Heavy Power** | 6 | 4.5 | 4.29 | 5.03 | 4.41 | 3.94 | 3.59 | 3.82 | 3.28 | 2.91 |
| **Heavy Lateral** | 5.69 | 6.28 | 5.12 | 5.16 | 4.97 | 4.53 | 3.22 | 4.09 | 3.81 | 3.46 |
| **Heavy Tip** | 5.94 | 9.59 | 7.72 | 5.91 | 5.28 | 5.28 | 4.19 | 4.22 | 3.09 | 3.1 |
| **Heavy Extension** | 7 | 11.84 | 8.19 | 12.09 | 5.63 | 5.97 | 4.28 | 9.53 | 3 | 3.09 |
| **Carton** | 41.81 | 18.09 | 21.25 | 20.31 | 17.82 | 16.53 | 12.84 | 14.37 | 11.28 | 12.38 |
| **Button** | 27.78 | 28.03 | 16.46 | 18.19 | 15.31 | 14.09 | 21.53 | 17.43 | 23.44 | 20.38 |
| **Heavy** | 17 | 4.84 | 6.03 | 6.53 | 4.62 | 4.25 | 3.91 | 3.96 | 2.66 | 2.75 |
| **Light** | 4.72 | 5.53 | 4.5 | 5.82 | 4.06 | 5.16 | 3.31 | 3.88 | 2.34 | 3.12 |
| **Screw** | 24.79 | 12.94 | 15.12 | 17.53 | 13.25 | 10.09 | 8.5 | 10.15 | 9.38 | DNF |
| **Handle** | 5.56 | 4.31 | 3.72 | 4.28 | 3.12 | 1.54 | 1.35 | 1.53 | 1.28 | 1.19 |
| **Jug** | 24.87 | 19.5 | 21.34 | 14.87 | 14 | 15.98 | 8.53 | 14.72 | 10.65 | 11.34 |
| **Key** | 6.09 | 4.69 | 5.63 | 4.53 | DNF | 4.03 | 3.03 | 6.66 | 3.63 | 10.94 |
| **Zip** | 10.12 | 9.75 | 6.84 | 8.21 | 4.47 | 6.05 | 3.56 | 4.03 | 5.03 | 2.56 |
| **Page** | 6.78 | 5.66 | 5.69 | 6.19 | 4.81 | 4.5 | 3.06 | 3 | 2.94 | 2.63 |

**Table S2 - mSHAP Results Subject 2**

Succesful completion times (s) for each task of the mSHAP for each session for Subject 2. DNF recorded for unsuccessful attempts

| Tasks | Session 1 | Session 2 | Session 3 | Session 4 | Session 5 | Session 6 | Session 7 | Session 8 | Session 9 | Session 10 |
|---|---|---|---|---|---|---|---|---|---|---|
| **Light Spherical** | 4.43 | 5.47 | 4.54 | 3.62 | 3.5 | 3.87 | 3.43 | 3.06 | 2.87 | 3.15 |
| **Light Tripod** | 7.22 | 5.85 | 6.34 | 4.94 | 3.66 | 5.53 | 4.66 | 4.13 | 4.91 | 3.22 |
| **Light Power** | 5.84 | 5.37 | 5.53 | 5.12 | 3.25 | 3.25 | 2.94 | 3 | 3.09 | 2.93 |
| **Light Lateral** | 5.34 | 6.5 | 5.19 | 5.06 | 3.28 | 6 | 4.68 | 3.59 | 2.85 | 4.29 |
| **Light Tip** | DNF | 7.94 | 7.72 | 4.56 | 4.28 | 4.78 | 3.59 | 3.75 | 4.22 | 5.06 |
| **Light Extension** | 14.28 | 7.75 | 5.22 | 5.65 | 3.37 | 4.97 | 4.22 | 3.19 | 4.09 | 4.47 |
| **Heavy Spherical** | 5.75 | 5.31 | 6.65 | 7.87 | 4.12 | DNF | 3.43 | 3.25 | 4.62 | 4.81 |
| **Heavy Tripod** | 7.78 | 13.75 | 6.13 | 5.31 | 4.81 | 4.69 | 5.47 | DNF | 8.71 | 3.82 |
| **Heavy Power** | 6.56 | 5.07 | 3.91 | 5.63 | 2.91 | 4.07 | 3.37 | 3.44 | 4.09 | 4.16 |
| **Heavy Lateral** | 6.31 | 8 | 4.56 | 4.5 | 3.34 | 3.84 | 4.34 | 3.19 | 3.69 | 3.56 |
| **Heavy Tip** | 8.78 | 7.03 | 4.72 | 4.03 | 4.22 | 10 | 5.87 | 4 | 3.85 | 3.78 |
| **Heavy Extension** | 6.09 | 5.91 | 5.62 | 4.78 | 4.28 | 4.09 | 3.97 | 4.94 | 4.68 | 4.07 |
| **Carton** | 22.72 | 19.53 | 20.25 | 19.69 | 14.87 | 18.78 | 13.81 | 19.06 | 16.38 | 16.38 |
| **Button** | 54.25 | 23.19 | 39.06 | 31.88 | 14.59 | 22.18 | 11.68 | 15.53 | 12.41 | 35.1 |
| **Heavy** | 7.06 | 6.69 | 5.43 | 4.22 | 5.56 | 6.03 | 4.96 | 5.06 | 5.37 | 5 |
| **Light** | 4.97 | 5.41 | 4.69 | 4.28 | 3.69 | 3.72 | 3.22 | 3.03 | 3.1 | 3.97 |
| **Screw** | 14.44 | 10.85 | 7.04 | 8.96 | 6.47 | 7.47 | 7.34 | 6 | 23.41 | 7.68 |
| **Handle** | 4 | 2.94 | 2.53 | 1.84 | 1.72 | 1.84 | 1.78 | 1.78 | 1.9 | 1.6 |
| **Jug** | 21.53 | 20.38 | 16 | 16.19 | 16.91 | 15.28 | 10.72 | 10.6 | 12.37 | 12.66 |
| **Key** | 4.53 | 3.56 | 3.03 | 3.9 | 4.12 | 3.4 | 3 | 2.03 | 2.4 | 2.71 |
| **Zip** | 6.47 | 5.88 | 4.16 | 5.03 | 7.41 | 5.35 | 4.53 | 9.03 | 11.09 | 6.31 |
| **Page** | 5.25 | 6.06 | 4.75 | 3.85 | 4.12 | 3.84 | 2.72 | 4.1 | 2.84 | DNF |

**Table S3 - mSHAP Results Subject 3**

Succesful completion times (s) for each task of the mSHAP for each session for Subject 3. DNF recorded for unsuccessful attempts

| Tasks | Session 1 | Session 2 | Session 3 | Session 4 | Session 5 | Session 6 | Session 7 | Session 8 | Session 9 | Session 10 |
|---|---|---|---|---|---|---|---|---|---|---|
| **Light Spherical** | 6.91 | 6.16 | 5.56 | 8.81 | 5.6 | 4.78 | 4.84 | 5.13 | 4.81 | 4.75 |
| **Light Tripod** | 9.19 | 8.6 | 5.5 | 5.9 | 7.22 | 7.71 | 6.03 | 6.53 | 6.09 | 5.5 |
| **Light Power** | 6.25 | 8.65 | 5.34 | 5.94 | 5.84 | 5.94 | 4.94 | 4.91 | 4.84 | 4.03 |
| **Light Lateral** | 8.12 | 6.62 | 5.66 | 5.28 | 5.41 | 7.44 | 4.85 | 5.81 | 4.9 | 4.57 |
| **Light Tip** | 9.5 | 9.04 | 9.5 | 6.15 | 5.19 | 7.41 | 8.06 | 5.91 | 6.53 | 7.34 |
| **Light Extension** | 14.31 | 10.79 | 6.35 | 5.31 | 6.13 | 7.25 | 6.9 | 6.91 | 6.53 | 5.5 |
| **Heavy Spherical** | 9.66 | 7.16 | 5.5 | 8.9 | 7.16 | 5.78 | 5.75 | 5.91 | 5.78 | 5.13 |
| **Heavy Tripod** | 8.71 | 8.03 | 7.75 | 6.25 | 8.87 | 10.29 | 13.1 | 5.66 | 6.78 | 5.75 |
| **Heavy Power** | 6.85 | 6.75 | 4.97 | 5.1 | 4.94 | 5.56 | 6.75 | 5.42 | 5.09 | 4.25 |
| **Heavy Lateral** | 8.35 | 5.88 | 6.19 | 5.56 | 4.94 | 8.5 | 4.93 | 10.56 | 5.4 | 4.4 |
| **Heavy Tip** | 6.81 | 11.59 | 8.25 | 6.6 | 5.31 | 7.88 | 6.97 | 5.53 | 6.72 | 5.44 |
| **Heavy Extension** | 7.06 | 7.85 | 8.43 | 4.78 | 6.12 | 6.68 | 8.03 | 8.43 | 7.62 | 5.5 |
| **Carton** | 31.15 | 19.8 | 19.16 | 18.88 | 16.85 | 18.25 | 17.53 | 15.25 | 18.38 | 17.56 |
| **Button** | 80.22 | 41.65 | 26.82 | 29.16 | 53.94 | 26.69 | 35.03 | 37.46 | 29.9 | 29.15 |
| **Heavy** | 6.78 | 5.53 | 4.85 | 5.19 | 5.41 | 5.59 | 6.28 | 4.59 | 5.19 | 4.75 |
| **Light** | 6.5 | 5.81 | 5.25 | 5 | 4.15 | 6.28 | 5.84 | 6.06 | 9.22 | 5.06 |
| **Screw** | 24.6 | 15.88 | 32.62 | DNF | DNF | 22.37 | 13.1 | 14.34 | 12.07 | 11.88 |
| **Handle** | 8.97 | 5.68 | 5.37 | 4.16 | 4.28 | 6.66 | 4.78 | 5.35 | 6.9 | 4.13 |
| **Jug** | 31.53 | 23.38 | 18.56 | 23.94 | 18.38 | 26.03 | 23.56 | 23.13 | 27.25 | 19.75 |
| **Key** | 8.79 | 7.44 | 6.16 | 7.23 | 8.16 | 7.35 | 8.06 | 6.84 | 8.81 | 6.87 |
| **Zip** | 9.56 | 6.53 | 6.28 | 5.87 | 6.47 | 7.66 | 6.97 | 14.97 | 6.81 | 5.72 |
| **Page** | 6.94 | 8.18 | 7.09 | 5.65 | 5.72 | 7.12 | 7.81 | 6.25 | 6.07 | 7.44 |

**Table S4 - mSHAP Results Subject 4**

Succesful completion times (s) for each task of the mSHAP for each session for Subject 4. DNF recorded for unsuccessful attempts

| Tasks | Session 1 | Session 2 | Session 3 | Session 4 | Session 5 | Session 6 | Session 7 | Session 8 | Session 9 | Session 10 |
|---|---|---|---|---|---|---|---|---|---|---|
| **Light Spherical** | 4.22 | 5.13 | 4.68 | 4.19 | 3.63 | 3.19 | 2.44 | 2.66 | 3.29 | 2.31 |
| **Light Tripod** | 5.31 | 3.94 | 6.97 | 3.69 | 6 | DNF | 3.91 | 3.34 | 5.37 | 4.04 |
| **Light Power** | 4.06 | 3.66 | 3.9 | 3.56 | 3.47 | 3.91 | 2.81 | 4.72 | 3.13 | 2.93 |
| **Light Lateral** | 4.06 | 4.28 | 5.47 | 5.82 | 4.41 | 3.91 | 3.84 | 3.25 | 3.97 | 4.06 |
| **Light Tip** | 5.47 | 5.34 | 4.28 | 4.31 | 7 | 3.75 | 3.5 | 3.72 | 3.81 | 4.47 |
| **Light Extension** | 5.97 | 5.41 | 7.19 | 5.16 | 4.78 | 3.91 | DNF | 4.65 | 6.69 | 4.03 |
| **Heavy Spherical** | 6.16 | 5 | 14.63 | 8.81 | 5.03 | 3.54 | 2.91 | 3.75 | DNF | 3.6 |
| **Heavy Tripod** | 4.9 | 3.94 | 4.37 | 3.37 | 4.34 | 3.61 | 6.93 | 3.09 | 4.32 | 3.34 |
| **Heavy Power** | 4.72 | 6.1 | 3.72 | 3 | 3.57 | 2.56 | 3.38 | 4.72 | 3.37 | 3.75 |
| **Heavy Lateral** | 4.66 | 5.06 | 4.31 | 5.19 | 4.38 | 11.19 | 3.9 | 3.1 | 3.37 | 9.12 |
| **Heavy Tip** | 7.22 | 5.56 | 5.9 | 3.57 | 4.25 | 3.28 | 4.93 | 3.09 | 4.6 | 4.85 |
| **Heavy Extension** | 4.35 | 5.87 | 5.59 | 4.69 | 4.22 | 5.13 | 4.09 | 3.28 | 3.91 | 3.38 |
| **Carton** | 16.97 | 15.5 | 17.04 | 13.97 | 14.81 | 11.38 | 9.94 | 11.03 | 11.2 | DNF |
| **Button** | 32.44 | 18.12 | 17.06 | 8.47 | 17.5 | 10.97 | 14.03 | 13.81 | 9.13 | 11.22 |
| **Heavy** | 7.34 | 4.09 | 4.72 | 5.06 | 4.78 | 4.25 | 2.84 | 3 | 3.34 | 3.25 |
| **Light** | 5.94 | 4 | 4.88 | 3.22 | 4.87 | 3.19 | 3.03 | 2.56 | 3.03 | 3.06 |
| **Screw** | 15.78 | 10.16 | 9.12 | 8.16 | 8.69 | 6.28 | DNF | 7.19 | 12.03 | 9.47 |
| **Handle** | 2.53 | 2.34 | 3.28 | 2.47 | 2.28 | 1.66 | 1.78 | 1.28 | 1.93 | 1.44 |
| **Jug** | 8.88 | 9.25 | 10.31 | 11.72 | 13.31 | 17.13 | 8.22 | 9.07 | 10.19 | 11.69 |
| **Key** | 4.03 | 3.91 | 3.21 | 3.19 | 2.96 | 3.25 | 2.94 | 11.26 | 5.94 | 4.94 |
| **Zip** | 7.16 | 5.25 | 7.72 | 4.32 | 5.62 | 4.5 | 11 | 6.57 | 16.81 | 5.15 |
| **Page** | 13.97 | 3.75 | 5.81 | 3.87 | 3.97 | 4.5 | 6.82 | 3.03 | 3.16 | 4.6 |

**Table S5 - mSHAP Results Subject 5**

Succesful completion times (s) for each task of the mSHAP for each session for Subject 5. DNF recorded for unsuccessful attempts

| Tasks | Session 1 | Session 2 | Session 3 | Session 4 | Session 5 | Session 6 | Session 7 | Session 8 | Session 9 | Session 10 |
|---|---|---|---|---|---|---|---|---|---|---|
| **Light Spherical** | 3.47 | 4.21 | 4.56 | 2.96 | 2.78 | 2.31 | 2.28 | 2.43 | 2.41 | 2.57 |
| **Light Tripod** | 4 | 4.84 | DNF | 3.25 | 3.75 | 4.19 | 3.63 | 4.25 | 3.53 | 3.62 |
| **Light Power** | 6.19 | 4.57 | 3.84 | 2.97 | 3.75 | 2.75 | 2.69 | 2.79 | 3.03 | 2.69 |
| **Light Lateral** | 3.59 | 5.22 | 5.75 | 4 | 3.65 | 2.63 | 3.25 | 2.6 | 2.75 | 2.63 |
| **Light Tip** | 4.15 | 5.6 | 7.12 | 9.38 | 4.34 | 5.72 | 3.91 | 3.66 | 5.15 | 5.06 |
| **Light Extension** | 5.66 | 8.84 | 3.87 | 4.06 | 3.94 | 3.66 | 6.65 | 3.93 | 3.88 | 4.82 |
| **Heavy Spherical** | 4.03 | 4.78 | 4.22 | 3.29 | 3.1 | 2.5 | 2.65 | 2.91 | 3.46 | 3.07 |
| **Heavy Tripod** | 4.31 | 5 | 6.53 | 3.22 | 4.03 | 3.62 | 3.81 | 4.56 | 3.5 | 3.37 |
| **Heavy Power** | 3.78 | 5.82 | 5.44 | 3.54 | 3.53 | 2.78 | 2.94 | 3.21 | 2.75 | 2.87 |
| **Heavy Lateral** | 7.65 | DNF | 4.31 | 4.5 | 3.66 | 2.9 | 2.75 | 3.62 | 3.16 | 3.22 |
| **Heavy Tip** | 4.72 | 5.69 | 4.16 | 4.34 | 3.63 | DNF | 3.1 | 4.5 | 3.5 | 3.44 |
| **Heavy Extension** | 4.34 | 5.06 | 4.37 | 3.81 | 4 | 3.78 | 3.44 | 3.78 | 3.97 | 9.75 |
| **Carton** | 17.88 | 16.03 | 18.78 | DNF | 20.37 | 15.5 | 13.56 | 16.06 | 16.38 | 16.69 |
| **Button** | 13.5 | 13.56 | 14.07 | 14.62 | 24.31 | 8.88 | 12.47 | 10.5 | 10 | 8.25 |
| **Heavy** | 7.28 | 4.25 | 3.68 | 3.69 | 3.5 | 2.47 | 2.81 | 3.1 | 3.09 | 3.09 |
| **Light** | 4.12 | 4.38 | 3.66 | 4.47 | 3.35 | 2.56 | 2.71 | 2.66 | 4.5 | 4.06 |
| **Screw** | 8.97 | 11.66 | 10.72 | 11.25 | 7.34 | DNF | 8.1 | 6.97 | 7.59 | 10.21 |
| **Handle** | 2.07 | 1.93 | 1.09 | 0.91 | 1 | 0.81 | 1.09 | 1.03 | 1.16 | 2 |
| **Jug** | 21.44 | 13.31 | 14.81 | DNF | 11.53 | 13.91 | 12.5 | 10.25 | 20.5 | 12.22 |
| **Key** | 6.1 | 6.56 | 4.25 | 3.25 | 5.84 | 2.57 | 2.97 | 3.03 | 3.03 | 3.37 |
| **Zip** | DNF | 10.09 | 6.72 | 5.44 | 7.25 | 5.21 | 5.62 | 4.06 | 4.41 | 4.13 |
| **Page** | 4.97 | 5.06 | 3.87 | 3.35 | 3.25 | 3.25 | 7.1 | 2.63 | 3 | 3.62 |

**Table S6 - mSHAP Results Subject 6**

Succesful completion times (s) for each task of the mSHAP for each session for Subject 6. DNF recorded for unsuccessful attempts

| Tasks | Session 1 | Session 2 | Session 3 | Session 4 | Session 5 | Session 6 | Session 7 | Session 8 | Session 9 | Session 10 |
|---|---|---|---|---|---|---|---|---|---|---|
| **Light Spherical** | 4.4 | 4.62 | 2.87 | 2.5 | 2.88 | 2.84 | 2.66 | 3.35 | 1.78 | 1.97 |
| **Light Tripod** | 4.9 | 11.12 | 2.66 | 2.5 | 3.09 | 3.63 | 3.41 | 2.97 | 2.87 | 2.9 |
| **Light Power** | DNF | 3.81 | 3.44 | 2.41 | 3.65 | 3.25 | 3.44 | 3.41 | 2 | 2.1 |
| **Light Lateral** | 3.57 | 5.41 | 4.25 | 3.12 | 3.34 | 4.12 | 2.82 | 2.49 | 3.28 | 2.13 |
| **Light Tip** | 5.79 | 5.56 | 4.6 | 2.78 | 5.39 | DNF | 3.18 | 3.84 | 3.06 | 2.81 |
| **Light Extension** | 5.41 | 4.75 | 3.4 | 4.47 | 3.69 | 4.15 | 4.03 | 5.47 | 4.22 | 3.1 |
| **Heavy Spherical** | 4.47 | 4.5 | 2.75 | 2.59 | 2.44 | 4.19 | 2.9 | 3.1 | 2 | 2.1 |
| **Heavy Tripod** | 5.35 | 3.6 | 4.47 | 3.09 | 3.4 | 4.31 | 4.03 | 3.37 | 2.34 | 2.72 |
| **Heavy Power** | 3.69 | 3.31 | 3.16 | 3.09 | 2.69 | 2.96 | 3.59 | 2.81 | 2.54 | 2.66 |
| **Heavy Lateral** | 4.19 | 3.75 | 2.75 | 2.56 | 8.06 | 5.28 | 3.88 | 8.82 | 3.12 | DNF |
| **Heavy Tip** | 5.1 | 4.94 | 3.19 | 2.84 | 2.87 | 2.81 | 3.06 | 3.72 | 2.43 | DNF |
| **Heavy Extension** | 6.88 | 4.53 | 3.87 | 4.31 | 5.69 | 3.87 | 3.58 | 2.97 | 3.13 | 3.59 |
| **Carton** | DNF | 14.38 | 13.63 | 14.69 | 14.05 | 13 | 15.78 | 13.94 | 11.37 | 13.5 |
| **Button** | 26.59 | 18.06 | 9.78 | 18.18 | 21.93 | 15.61 | 15.53 | 16.25 | 9 | 10.03 |
| **Heavy** | 3.84 | 2.6 | 2.63 | 2.53 | 3.19 | 3.91 | 3.34 | 4.25 | 2.53 | 3.69 |
| **Light** | 3.21 | 2.91 | 2.43 | 3.97 | 2.5 | DNF | 5.29 | 2.94 | 2.47 | 3.1 |
| **Screw** | 9.41 | 8.35 | 13.63 | DNF | 9.59 | 18.65 | 5.29 | 7.34 | 7.07 | 6.75 |
| **Handle** | 3.45 | 2.13 | 2.56 | 3.25 | 2.09 | 2.72 | 2.13 | 2.66 | 1.44 | 3.12 |
| **Jug** | 19.28 | 9.38 | 10.22 | 8.5 | 10.16 | 9.63 | 9.38 | 9.25 | 6.47 | 8.41 |
| **Key** | 3.84 | 4.43 | 3.34 | 2.28 | 2.37 | 2.81 | 2.31 | 2.75 | 2.31 | 2 |
| **Zip** | 4.56 | 3.97 | 5.82 | 2.47 | 2.65 | 7.21 | 5.32 | 6.15 | 6.53 | 3.81 |
| **Page** | 8.37 | 3.16 | 2.69 | 2.25 | 2.97 | 2.94 | 2.47 | 2.81 | 1.88 | 3.31 |

**Table S7 - BBT Results**

Number of blocks successfully transported in each BBT trial for each session ( 3 trials per session) and subject

| Subject | Session 2 | Session 3 | Session 4 | Session 5 | Session 6 | Session 7 | Session 8 | Session 9 | Session 10 |
|---|---|---|---|---|---|---|---|---|---|
| **1** | N/A | N/A | 17 | 20 | 31 | 32 | 37 | 40 | 43 |
| | N/A | N/A | 18 | 25 | 32 | 41 | 45 | 40 | 47 |
| | N/A | N/A | 16 | 23 | 36 | 41 | 44 | 46 | 38 |
| **2** | N/A | N/A | 24 | 32 | 31 | 32 | 35 | 26 | 35 |
| | N/A | N/A | 30 | 31 | 38 | 33 | 36 | 35 | 42 |
| | N/A | N/A | 27 | 33 | 32 | 28 | 37 | 33 | 40 |
| **3** | 15 | 21 | 29 | 34 | 28 | 25 | 30 | 34 | 30 |
| | 20 | 23 | 29 | 31 | 27 | 24 | 29 | 31 | 30 |
| | 20 | 24 | 28 | 27 | 31 | 27 | 34 | 31 | 32 |
| **4** | 25 | 24 | 25 | 28 | 37 | 35 | 39 | 36 | 40 |
| | 29 | 29 | 25 | 29 | 30 | 35 | 34 | 37 | 41 |
| | 25 | 30 | 32 | 31 | 35 | 34 | 40 | 38 | 42 |
| **5** | 21 | 23 | 29 | 27 | 33 | 33 | 30 | 32 | 42 |
| | 20 | 24 | 30 | 34 | 36 | 36 | 44 | 35 | 38 |
| | 22 | 28 | 30 | 30 | 33 | 34 | 34 | 36 | 42 |
| **6** | 27 | 33 | 34 | 30 | 36 | 36 | 48 | 48 | 43 |
| | 30 | 36 | 29 | 31 | 39 | 47 | 41 | 47 | 34 |
| | 32 | 34 | 37 | 33 | 37 | 43 | 48 | 47 | 44 |

**Table S8 - Number of Quadrant Changes during mSHAP (Prepositioning)**

Number of times subjects changed quadrants (orientation of the terminal device) across all 22 tasks of the mSHAP

| Subject | Session 1 | Session 2 | Session 3 | Session 4 | Session 5 | Session 6 | Session 7 | Session 8 | Session 9 | Session 10 |
|---|---|---|---|---|---|---|---|---|---|---|
| **1** | 8 | 11 | 6 | 3 | 4 | 10 | 7 | 12 | 11 | 9 |
| **2** | 5 | 9 | 12 | 11 | 14 | 13 | 15 | 13 | 10 | 10 |
| **3** | 8 | 10 | 10 | 5 | 6 | 9 | 5 | 5 | 3 | 4 |
| **4** | 7 | 3 | 6 | 9 | 4 | 8 | 6 | 4 | 4 | 5 |
| **5** | 6 | 13 | 7 | 9 | 6 | 8 | 10 | 5 | 8 | 6 |
| **6** | 4 | 5 | 8 | 10 | 5 | 9 | 8 | 8 | 6 | 8 |
